# The hematopoietic tissue of the freshwater crayfish, *Pacifastacus leniusculus*: organization and expression analysis

**DOI:** 10.1007/s00441-024-03943-1

**Published:** 2025-01-04

**Authors:** Thanapong Kruangkum, Kenneth Söderhäll, Irene Söderhäll

**Affiliations:** 1 Department of Organismal Biology, Uppsala University, Norbyvägen 18A, 75236 Uppsala, Sweden; 2 Department of Anatomy, Faculty of Science, Mahidol University, Bangkok, Thailand; 3 Center of Excellence for Shrimp Molecular Biology and Biotechnology (CENTEX Shrimp), Faculty of Science, Mahidol University, Bangkok, Thailand

**Keywords:** Hematopoietic tissue, Hematopoiesis, Crayfish, Hemocyte, Cell proliferation

## Abstract

The hematopoietic tissue (HPT) and anterior proliferation center (APC) are the main hemocyte-producing organs of the freshwater crayfish, *Pacifastacus leniusculus*. To deepen our understanding of immune responses to various pathogens, it is essential to identify distinct hemocyte subpopulations with specific functions and to further explore how these cells are generated. Here we provide an in-depth histological study of the HPT and APC in order to localize cell types in different developmental stages, and to provide some information regarding the hemocyte differentiation in the crayfish. We localized mRNA expression of previously identified genes in the HPT/APC and hemocytes by RNA-FISH. The expression of hemolectin and transglutaminase 1 was shown to be co-localized in a high number of the HPT cells, while transglutaminase 2 was expressed in different cell types mainly associated with epithelium or endothelium. Furthermore, by double RNA-FISH for hemolectin and a previously unidentified PDGF-like factor, combined with immunostaining for prophenoloxidase, we could identify several different subtypes of hemocytes, indicating that the immune function of hemocytes in crayfish is more diversified and complex than previously appreciated.

**Supplementary Information:**

The online version contains supplementary material available at 10.1007/s00441-024-03943-1.

## Introduction

Hematopoietic organs play a major role in immune systems by providing a source for hemocyte production and their release into the free circulation (Grigorian and Hartenstein 2013; Söderhäll 2016; Rosental et al. 2020; Elsaid et al. 2020; Söderhäll and Söderhäll 2022). The hematopoietic tissue (HPT) is often localized on the stomach surface, and is the hemocyte producing tissue in crustaceans, and has been described in detail in crustacean species by a few research groups (Söderhäll et al. 2003; Söderhäll 2016; Söderhäll and Söderhäll 2022). During the last decades, some progress has been made to uncover the mechanisms behind hemocyte formation, differentiation, and reactions responding to invasive pathogens as well as the HPT as a source for progenitor cell differentiation to other cell linages, e.g., neuronal progenitor cells (Cerenius and Söderhäll 2018; Liu et al. 2021; Benton et al. 2022; Söderhäll et al. 2022). However, the precise differentiation pathways to different cell types are still not fully clarified.

In freshwater crayfish (*Pacifastacus leniusculus*), the morphology and localization of HPT have been well described and it is structurally organized in a thin translucent sheath covering the roof of the stomach. It contains a large number of lobules of hemocyte precursor cells, which was shown by the use of light and transmission electron microscopy (Chaga et al. 1995; Söderhäll 2016). In the HPT there is a high activity of cell division demonstrated by BrdU incorporation assays, and it is especially high in the anterior proliferation center (APC) of the hematopoietic organ. The APC connects and locates anteriorly to the HPT and the cell proliferation is very high in conjunction with a high production of reactive oxygen species (ROS) (Noonin et al. 2012; Chaves Da Silva et al. 2013; Junkunlo et al. 2016; Benton et al. 2022).

Recently, the hematopoietic organ (APC + HPT) and hemocytes (HCs) of *P. leniusculus* were categorized into 14 clusters based on the differential expression of the dominant transcripts by single cell transcriptome analysis (Söderhäll et al. 2022). Interestingly, this study showed that some specific marker genes were expressed in different hemocyte clusters of both HPT and HCs. With this technique, a new phase in cell classification of crayfish hemocytes and HPT was made possible, instead of only by morphological criteria, and will be essential to understand the mechanism of crayfish and crustacean hematopoiesis.

The morphology of the HPT and the APC has been well described as mentioned above. However, the histological organization, cell populations in the lobules, as well as specific localized gene expression are in need of a more detailed analysis, since classification by the morphology and ultrastructure by light and electron microscopies is not fully efficient for a complete determination of hemocyte types and subtypes.

In situ hybridization by RNA-FISH by fluorescent detection is a powerful technique for transcript localization in cells and tissues, and this method was used in a previous study in the isolated HPT cells and hemocytes (Söderhäll et al. 2022). It provided details about the type of cells which expressed some of the marker genes, but still further studies are required to obtain a more complete picture of the hemocyte clusters and types in HPT and circulation.

The aims of the present study were 1) to investigate the histology, cellular organization, and proliferation of cells in HPT and APC using routine H&E staining and BrdU incorporation assays; and 2) to reveal the distribution of expressed genes associated with hematopoiesis and differentiation in the HPT, APC, and HCs of crayfish using RNA-FISH as well as immunofluorescent staining.

## Materials and methods

### Animals

Freshwater crayfish, *P. leniusculus*, were caught in and brought from Lake Erken, Sweden. The animals were housed in aquaria containing running aerated tap water, with controlled ambient temperature around 12 °C. Only intermolt animals, and animals with no visible injuries were used in the experiments.

### Indian-ink administration for vascular labeling

Male crayfish (carapace length: 3–3.5 cm) were submersed in an ice-basket for 10 min and then India-ink (around 100–200 μL, Pébéo, France) was gently introduced into the heart using a 1-mL syringe with a 27G^¾^ needle. Then the dorsal carapace was carefully removed from the animals, while the lining epidermis was left intact. After injection for 2–3 min, the animals were fixed immediately in 4% paraformaldehyde fixative solution (PFA) pH 7.4 and kept at 4 °C overnight. The tissues were then washed with 1 × phosphate buffered saline (PBS, containing 137 mM NaCl, 2.7 mM KCl, 8 mM Na_2_HPO_4_, and 2 mM KH_2_PO_4_, pH 7.4) at least five times before dissection. The tissues were dissected under a stereomicroscope (Nikon SMZ1500) and were photographed using a digital camera (NiKon DS-Vi1). The epidermis covering internal organs was gently removed. The dorsal surface of the stomach, which is the site for the hematopoietic tissue (HPT), was dissected from the animal for further processing. The connective tissue containing the anterior proliferation center (APC) was also dissected still attached to the brain.

### Tissue collection and preparation

The HPT attached with a dorsal plate of the stomach and the APC linked with the brain were dissected from the animals after paraformaldehyde fixation. The HPT-tissues prepared for sectioning in the coronal plane were pre-embedded in 1.5% agarose with 1 × PBS to maintain their shape before further processing. The samples were placed into tissue cassettes for passing through dehydration, clearing, and paraffin infiltration. Briefly the tissue cassettes were dehydrated by an increasing serial concentration of ethanol (70 to 100%), and then cleared twice by xylene immersion. They were then infiltrated with melted Paraplast (HistoLab, Sweden) overnight before embedded into paraffin blocks for further use. The rigid tissue block was trimmed and cut into serial thin tissue sections of 5- to 6-µm thickness using a rotary microtome (Leica RM2155). Coronal, sagittal, and horizontal planes of the HPT tissues and horizontal and sagittal sections of the APC were attached to Superfrost glass slides before further use in histological or immunofluorescence staining and RNA fluorescence in situ hybridization (RNA-FISH).

### Hematoxylin and eosin (H&E) staining

Paraffin-sectioned tissues were deparaffinized by immersion into xylene three times. The samples were then rehydrated by an ethanol series from 100 to 70%, and then moved to distilled water. For staining, the tissues were immersed in Mayer’s hematoxylin for 30 s before activating the colorization by running tap water for 2–3 min. The tissues were then transferred into eosin for 1 min before dehydration by a serial increasing concentration of ethanol (80 to 100%), and then subsequently cleared three times by xylene immersion. They were finally covered with a cover slip with VectaMount, permanent mounting medium (Vector Laboratories).

### Probe design and synthesis

Custom-branched probes for use with ViewRNA in situ hybridization assays (Invitrogen) were designed according to the sequences in Supplementary Table S1, and provided by ThermoFisher Scientific. The probes which were used in this study were targeting genes that can be categorized into four groups: (1) well known genes playing a role in hematopoiesis and immune response including hemolectin, transglutaminase 1 (TGase1), and transglutaminase 2 (TGase 2); (2) a melanization inhibition protein (MIP) and pacifastin heavy chain (pacifastin-HC) which were specific for two different small cell clusters in the HPT (Söderhäll et al. 2022); (3) previously unidentified genes discovered by transcriptomic analysis and believed to be involved in hematopoiesis and immune response, including endoglucanase (cenB) and PDGF- and VEGF-related factor 3 (PVF3); and lastly (4) E-cadherin was used to identify the endothelial and epithelial cells.

### RNA-fluorescence in situ hybridization in tissue sections

After deparaffinization and rehydration, all tissue sections (coronal and sagittal planes of HPT and APC) were processed based on the recommendation from the manual guide (ViewRNA™ Tissue Fluorescence Assay (Invitrogen)). Briefly, the tissue sections were treated with 1 × pretreatment solution at 90 °C for 10 min. They were immersed in a jar containing nuclease free water and 1 × PBS before treatment with a working protease solution (1:100 dilution in pre-warmed 1 × PBS) at 40 °C for 10 min in a temperature-controlled oven. They were then washed thrice with 1 × PBS before fixation with 4% PFA solution for 5 min at room temperature. They were then washed three times with 1 × PBS before probe hybridization. The target probe hybridization was performed (Supplementary Table S1) in pre-warmed probe set diluent QF in a dilution of 1:40 according to the assay manual. Tissue sections without target probes were used as negative controls. The hybridization was performed in a moisture chamber placed in a temperature-controlled oven at 40 °C for 2 h, and then washed three times with washing buffer. Preamplification and amplification were performed using a dilution of 1:25 in pre-warmed amplifier diluent QF for 30 min each step at 40 °C. The procedure for probe labeling was done according to the manufacturer’s user manual. After rinsing with washing buffer and 1 × PBS for three time each, the slides were counterstained with nuclear stain, DAPI (1:100 in 1 × PBS) for 10 min, or Hoechst 33258 in 1:1000 dilution in 1 × PBS for 20–30 min at room temperature. Interference of auto-fluorescence was eliminated by Ready Probe™ Tissue Autofluorescence Quenching Kit (Invitrogen, Thermo Fisher Scientific). The final washing was performed with 1 × PBS before mounting with ProLong™ Gold Antifade mounting medium (Invitrogen, Thermo Fisher Scientific), and overlaid with a cover slip. The tissue slides were kept in a slide box placed in a fridge until they were used for observation.

### Hemocyte collection and RNA-fluorescence in situ hybridization

The hemolymph of crayfish was collected by insertion of an 18-G needle (BD microlane) at the middle of the ventral area of the second abdominal segment of crayfish. Hemolymph was collected in 1:1 volume of anti-coagulant solution (0.14 M NaCl, 0.1 M glucose, 30 mM trisodium citrate, 26 mM citric acid, 10 mM EDTA, pH 4.6 (Söderhäll and Smith 1983). After centrifugation at 800 × g for 5 min at 4 °C, the hemocyte pellets were resuspended in 0.15 M NaCl solution. The cell suspension was carefully dropped onto Superfrost glass slides and mixed with 1 M CaCl_2_ solution in a ratio of 9:1. The cells were left for attachment for 30 min and fixed in 3.7% formaldehyde in crayfish saline (CFS, 0.2 M NaCl, 5.4 mM KCl, 10 mM CaCl_2_·2H_2_O, 2.6 mM MgCl_2_·6H_2_O, 2 mM NaHCO_3_, pH 6.8) for 10 min at room temperature. The attached cells on glass slides were immediately used for further procedures as described in the manual for QuantiGene ViewRNA ISH Cell assay kit (Invitrogen). The RNA-FISH protocol for cells follows the procedure above for tissue sections, but without any high-temperature pretreatment. The protocol was also modified and optimized for crustacean hemocytes. Briefly, the samples were treated with working protease solution (1:2000 dilution in 1 × PBS) at 40 °C for 15 min. The cell samples were washed with 1 × PBS before application of the specific probes according to Supplementary Table S1. The target specific probe dilutions were 1:100 in pre-warmed probe set diluent (QF) and incubated at 40 °C in a moisture chamber for 3 h. After this step, the samples were treated in a similar way as the tissue sections and were counterstained with a nuclear stain as described above. Then they were mounted with ProLong™ Gold Antifade mounting medium for further analysis.

### Cell proliferation assay with BrdU

Crayfish (carapace length: 3–3.5 cm) were injected with 10 μL of 50 mM BrdU in crayfish saline (CFS) per g of fresh crayfish body weight. After 20–24 h of BrdU incorporation, the crayfish were sacrificed for tissue collection. The dorsal carapace was carefully peeled back to allow penetration of 4% PFA fixative solution into the epidermis and internal organs immediately after fixation. The immersed cephalothorax in fixative solution was gently shaken at 4 °C for 24 h before washing. The HPT and APC were dissected from the animals and processed as mentioned above (“Tissue collection and preparation” section). The serial tissue sections were used for BrdU detection according to Noonin et al. (2012).

### BrdU incorporation and immunolocalization of TGase1 and pacifastin-HC

The coronal sections of HPT were deparaffinized and rehydrated as described above, before they were treated with 2 N HCl solution at 37 °C for 30 min. The sections were washed thrice with 1 × PBS and 0.3% PBST (0.3% triton-X100 in 1 × PBS) for 2 min each. Then they were incubated with blocking solution containing 5% bovine serum albumin (BSA) in 0.3% PBST for 40 min at room temperature. The primary antibody against BrdU produced in mouse (BD Bioscience) was diluted with 1:50 in blocking solution, applied to the tissue sections, and incubated in a moisture chamber at 37 °C for 2 h. The sections were washed thrice with 0.3% PBST for 3 min each before application with the secondary antibody (dilution 1:500 of goat anti-mouse IgG, conjugated-FITC, Invitrogen, USA, in blocking solution) for 2 h at room temperature in the dark. Negative controls without primary antibody were also done. After treatment with the secondary antibody, the tissues were gently washed with 0.3% PBST and 1 × PBS, 3 min each. Auto-fluorescence was eliminated by Ready Probe^TM^ Tissue Autofluorescence Quenching Kit (Invitrogen, Thermo Fisher Scientific) for 10 min at room temperature, followed by three times washing with 1 × PBS. Nuclear staining was performed with DAPI (1:100 in 1 × PBS) for 10 min at room temperature or Hoechst 33258 nuclear stain in 1:1000 dilution in 1 × PBS for 20–30 min at room temperature. After a final washing with 1 × PBS, the slides were treated with ProLong^TM^ Gold Antifade Mounting medium and overlaid with a cover slip for further observation.

Co-localization of proteins with BrdU incorporation was performed with an antibody against TGase1 or a pacifastin heavy chain (HC) antibody (Table 1) which had been purified on PVDF membranes against purified recombinant pacifastin heavy chain from *P. leniusculus*. The primary antibody solution was prepared by mixing the antibody against BrdU produced in mouse in a dilution of 1:50 and rabbit anti TGase1 (dilution 1:1000) or anti-pacifastin-HC (dilution 1:100) in blocking solution. The primary antibody and negative controls (without primary solution) were incubated with the tissues overnight in a moisture chamber at 4 °C.

**Table 1:** List of antibodies used in this study

Type	Name	Company	Cat/ref no	Reference
Primary Ab	Rabbit anti-proPO	-	-	Jearaphunt et al. (2014)
Primary Ab	Rabbit anti-giant freshwater prawn transglutaminase	-	-	Junkunlo et al. (2020)
Primary Ab	Mouse anti-BrdU	BD Bioscience	347580	-
Primary Ab	Rabbit anti-pacifastin-HC	-	-	Liang et al. (1997)
Secondary Ab	Goat anti mouse IgG (H + L), conjugated-FITC	Invitrogen	A16079	-
Secondary Ab	Goat anti-rabbit IgG, whole molecule FITC	Sigma	F0382	-
Secondary Ab	Goat anti-rabbit IgG Alexa 594	ThermoFisher Scientific	A-11012	-

### Co-localization with RNA-FISH and proPO antibody

Slides with hemocytes analyzed by RNA-FISH (probed with *Hml* and *PVF3*) were reused for additional immunostaining with antibody produced in rabbit against proPO recognizing the N-terminus of proPO (Jearaphunt et al. 2014) (Table 1). After washing the slides with 1 × PBS, they were treated with a blocking solution containing 5% normal goat serum in 1 × PBS for 40 min at room temperature. The samples were incubated with primary antibody diluted at 1:100 in blocking solution overnight at 4 °C. Slides without primary antibody were processed as a negative control. After overnight incubation, they were gently washed with 0.1% PBS containing tween-20 (washing buffer, viewRNA™ Tissue Fluorescence Assay, Invitrogen) 2 min each for three times. After washing, then incubation with secondary antibody (goat anti-rabbit IgG, whole molecule FITC, Sigma) diluted at 1:500 in 1 × PBS was performed for 2 h at room temperature. List of antibodies used are shown in Table 1. After gentle washing with the 0.3% PBST and 1 × PBS three times, 2 min each, the samples were mounted with ProLong™ Gold Antifade mounting medium for further observation.

### Microscopy and image analysis

H&E-stained tissue sections were observed under wide-field light microscope (Leica, DM5500B) and then visualized and photographed by LASX software (Leica Application Suite X). Fluorescence-stained tissue sections were observed under a wide-field microscope using fluorescent mode (Leica, DM5500B) by different filter channels: A4 (for DAPI detection), Y3 (for Alexa Flu 594 detection), L5 (for Alexa Flu 488 detection), and Y5 (for Alexa Flu 647 detection). In order to avoid artifacts due to fluorescence interference, samples labeled with Alexa Fluor 594 and Alexa Fluor 647 were observed using confocal laser scanning microscope (Leica Stellaris 5) and photographed using LASX software (Leica Application Suite X). Due to the close emission spectra of fluorophores Alexa Fluor 594 and 647, which both emit in similar shades of red, the emission signal of Alexa Fluor 594 was in some figures represented using a pseudo-color (green) to facilitate differential localization (*Hml* in Fig. 6). Multi-signal detection by confocal microscopy was done using a sequential mode to reduce the artifacts from the fluorescent crosstalk (fluorescent signal interference). The signal detection and exposure were adjusted based on the signal from the negative control.

The photographs were exported from the software before adjustment of brightness-contrast to obtain a better quality of photos using Adobe Photoshop 2024.

### Cell identification and classification

Differential phase-contrast (DIC) photos of hemocytes in three different areas from a stained slide were used for granular and semi-granular hemocyte classification and quantification. Only typical characteristics of hemocyte types were considered, while those with ambiguous characteristics were not included for calculation. After that the DIC images were merged with its exported colorized-image specific to transcript markers for categorization and measure based on the signal localization. Seven hemocyte types were classified as type I (high expression of proPO), type II (low expression of proPO), type III (co-expression of proPO and Hml), type IV (Hml only), type V (co-expression of PVF3 and Hml), type VI (PVF3 only), and type VII (clear cell, nuclear dominant no expression of used markers). Total cells (20–30 cells per whole area of each image) were counted individually for each image based on a presentation of nuclear marker (DAPI). Percentages of the types of hemocytes (types I–VII) after clear identification of “granular” or “semi-granular” groups were counted and measured from individual images, then combined with the data from three separate images for analysis. The percentages of hemocyte subtypes were represented separately for “granular” or “semi-granular” class.

## Results

### Anatomy and histology of the hematopoietic tissue

**Fig. 1:**
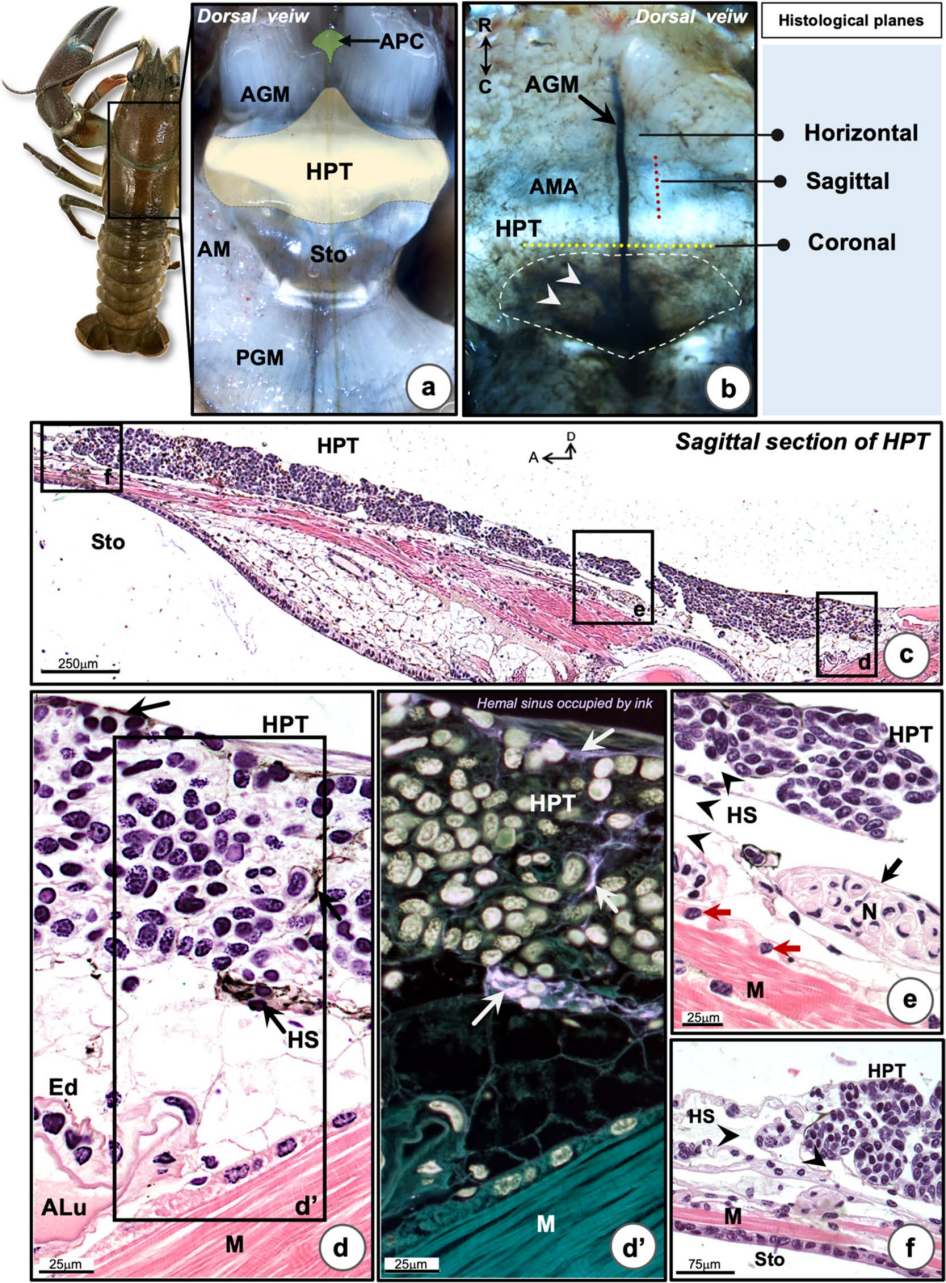
The morphology and histology of the hematopoietic tissue (HPT) of the freshwater crayfish, *P. leniusculus.* **a** Thin membrane of HPT is on the dorsal surface of the stomach (Sto) (yellow area). **b** India-ink signal after vascular injection was detected in the anterior median artery (AMA). The ink signal was also dispersed throughout the thin sheath area (indicated by white-dashed line area, arrow heads) nearby the inter-junction with the heart. The ink-labeled HPTs were used for histological studies. H&E staining in different planes; coronal, horizontal, and sagittal planes. **c** Low magnification of the sagittal HPT section after H&E staining. It is located and associated with the dorsal part of the stomach (Sto), while, the longitudinal muscle bundles (M) are found at the intermediate layer between both structures. **d** Indian-ink was present throughout the sinusoidal space of the HPT section (black arrows). (**d’**) An inverted dark field photograph showed the ink-labeling in the sinusoidal space (white arrows). **e** Histological analysis revealed a large sub-HPT hemal space (HS) located beneath the HPT (arrow heads) and this space is associated with the sub-muscular layer. The nerve bundle (N, arrow) found in this area could supply the HPT and/or the muscle bundles (M). **f** The margin of HPT is closely associated with the HS (arrow heads). AGM, anterior gastric muscle; PMG, posterior gastric muscle; R, rostal; C, caudal; D, dorsal; A, anterior; Ed, endothelium; ALu, arterial lumen; AM, adductor muscle

The hematopoietic tissue (HPT), which is a translucent membrane-like tissue, covers the dorsal part of the stomach of *P. leniusculus*. This tissue is located underneath the carapace (Fig. 1a). The HPT covers the epigastric area, anterior to the posterior margin of the anterior gastric muscle (AGM). At the anterior median, the HPT extends to connect with the anterior proliferation center (APC), which is located between the right and left AGMs and which spreads over the transvers gastric endo-skeletal ridge of the stomach (Fig. 1a). The HPT covers and extends only over the posterior part of the stomach, and not through the whole stomach. However, the posterior lateral part of HPT associates with the adductor muscle (Fig. 1a).

India ink administration was performed into the cardiac chamber and pericardial area for vascular labeling (Fig. 1b). An intense ink signal was observed inside the anterior median artery (AMA) which is located at the median part of the HPT. It runs anteriorly along the dorsal margin of the stomach. Some pale-ink signals were found dispersed around the HPT tissue, especially in the posterior part of the HPT (Fig. 1b).

In order to clarify the cellular structure of HPT, histological studies of the HPT in three anatomical orientations (sagittal, horizontal, and coronal planes) were done by a light microscope (Figs. 1c–f and S1). The parasagittal section of the HPT stained with H&E revealed an extensive clustering of HPT cells densely packed into several lobules, on top of the longitudinal muscular tissues, located on the top of the gastric-epithelial tissue (Fig. 1c). A highly magnified image of the posterior part of the HPT revealed a variety of cell-type organization in the HPT lobules. Eu- and hetero chromatic nuclei of HPT cells were identified inside the lobules (Fig. 1d). Labeling with ink indicated that it could penetrate into the HPT tissue and it was found superior, intermediate, and below the HPT lobules and was present at the site of sinusoidal space linked to the hemal space (Fig. 1d and d’, arrows). Underneath the HPT in the loose connective tissue, a site with vessels (Fig. 1d and d’) and nerves (Fig. 1e, black arrow) can be seen. The longitudinal muscle strings were observed to be embedded or enwrapped in the hemal space and lined with some cells of unknown function (Fig. 1e, red arrows). In the anterior part of HPT, there is a large hemal space, located at the same level of the HPT. This space may connect with the hemal space underneath the HPT by thin connective tissues (Fig. 1f, arrowheads).

The coronal HPT sections revealed that the anterior median artery (AMA) was found at the median part of HPT (Fig. S1a). In the lateral margin of HPT close to the lateral adductor muscle, the intact HPT tissues were shown to be associated with epidermis. There is a loose space located between the HPT and epidermis which is expected to be the hemal space (Fig S1b, b’). There were small vessels (arteries) and hemal sinus located in the interlobular space of the HPT tissue (Fig. S1b). Medial to the lateral HPT cluster, there is a large hemal sinus where mature hemocytes were found (Fig. S1b and b’). The AMA is filled with hemolymph and surrounded by a single layer of endothelial cells (Fig. S1c). A large hemal sinus is enveloped in the artery, called “periarterial sinus,” and it is continuously linked with the subHPT-hemal sinus (Fig. S1c). At the lateral part, there is a transverse artery present underneath of the HPT and the sub-hemal sinus (Fig. S1d). In the horizontal plan, the HPT tissue associates with the AMA (Fig. S1e). If higher magnification was employed, the HPT lobules were easily distinguished (Fig. S1f). A variety of different cell types, some undergoing mitoses, were identified inside the lobules (Fig. S1f; metaphase cell, yellow arrow; prophase cell, red arrow, and S1h; anaphase cells, yellow arrow head). The ink signal was found inside the arterial lumen, but no signal in the periarterial hemal sinus (Fig. S1g). The HPT cells undergoing mitosis, early mature hemocytes with condensed euchromatic nuclei (Fig. S1h), and large cells with euchromatic nuclei (Fig. S1i) were observed in each HPT lobule, while early mature hemocytes containing eosinophilic cytoplasm were found in the area immersed with ink-filled hemal sinuses (Fig. S1j).

### Localization, morphology and histology of the anterior proliferation center

**Fig. 2:**
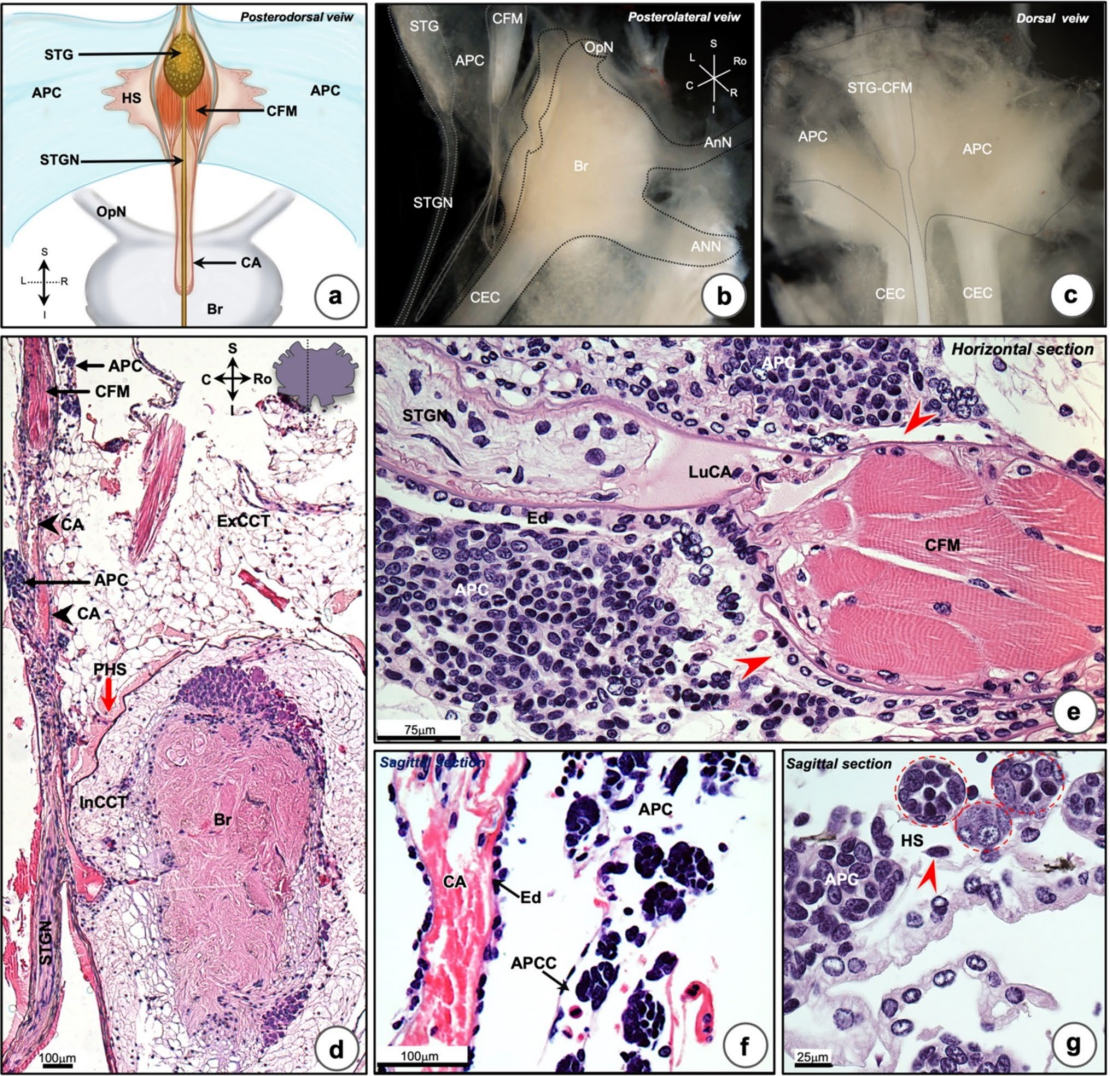
Localization, morphological and histological structures of the anterior proliferation center (APC). **a** An illustration of the location of APC associated with the brain (Br). The thin sheath of APC enwrapped in a pair of the cor frontale longitudinal muscle (CFM) and associated with the stomatogastric ganglion (STG) and nerve. **b**, **c** Stereomicroscopic photographs of the APC-Br complex observed from posterior-lateral and dorsal views, respectively. **d** A low magnification of sagittal section of APC-Br complex stained with H&E showed that the thin layer of APC is located and associated with the CFM and descending cerebral artery (CA, arrowheads). The pericerebral hemal space (PHS, red arrow) associated with the APC and the CA. **e** Horizontal section through the median part of APC, where the CFM is marked. The longitudinal bundles of CFM are organized inside the lumen of the cerebral artery (LuCA) where the STGN also was found. The APC tissues are located in the periphery to the CA, while the hemal sinus (HS) was observed associated with the CFM (red arrowheads). **f** A sagittal section of APC revealed that the APC lobules are located inside a thin layer of connective tissue connected to their APC canals (APCC), which is a site for mature hemocyte release. **g** Higher magnification of the APC showed the organization of APC lobules (red circles), which contain euchromatic and heterochromatic nuclei. Release of mature hemocyte was detected in the HS (red arrowhead). OpN, optic nerve; CEC, circumesophageal connecting; R, right; L, left; S, superior; I, inferior; Ro, rostral; C, caudal; Ed, endothelium; ExCCT, external cerebral connective tissue; InCCT, internal cerebral connective tissue; S, superior; I, inferior; R, rostral; C, caudal; STGN, stomatogastric nerve

The anterior proliferation center (APC) is localized anteriorly to the HPT. There are two straps of thin membrane, continuously extending from the lateral part of the right and left HPT. At the median part of APC, an association with the anterior extension of the AMA, which was composed of a pair of the cor frontale longitudinal muscles (CFM), the stomatogastric ganglion (STG), and its nerve (STGN) (Fig. 2a–c), was detected. The cerebral artery (CA) continued from the AMA before fusing with the brain sheath at the dorsal part. A parasagittal section of the APC-brain complex showed that the APC enclosed the cerebral artery (CA) at the dorsal and ventral sides (Fig. 2d). The CFM and STGN were identified and were found inside and associated with the lumen of the CA (Fig. 2d and e). Close to the junction of CA and brain, there is an area of brain-sheath enveloping the brain. The brain is surrounded firmly by a compact organization of external and internal cerebral connective tissues (ExCCT and InCCT, respectively). A large space, called “pericerebral hemal space,” was found between both these areas of connective tissue (Fig. 2d). Beside the cor frontale longitudinal muscles, tiny spaces were found (Fig. 2e). It was surrounded by APC cells (Fig. 2e). Noticeably, the APC is not as clearly organized into lobules as the HPT when observed in the horizontal plane of tissue close to the artery (Fig. 2e), although there are lobule-like structures or rosettes as described by Chaves da Silva et al. (2013). High magnification of the histology of the APC in the sagittal plan revealed an APC canal where the APC lobules and released mature hemocytes were detected (Fig. 2f). The histology of the APC lobules showed their organization as being circular to oval in shapes. The cells in APC lobules were in a variety of different mitotic stages (Fig. 2g, in the red dot circles).

Thus, we find no clear zones with lobules of different morphology within the HPT, instead there is an indication that cells of different developmental stages are localized within each individual lobule. Further, we confirm that the lobular structure is less organized within the APC, with the presence of some rosette-like structures. We also conclude that there seems to be no direct connection between the HPT and the AMA, and that HPT cells most likely are released into the hemal sinus.

### Expression of TGase mRNA (TGase 1 and 2) in the HPT and APC tissues

**Fig. 3:**
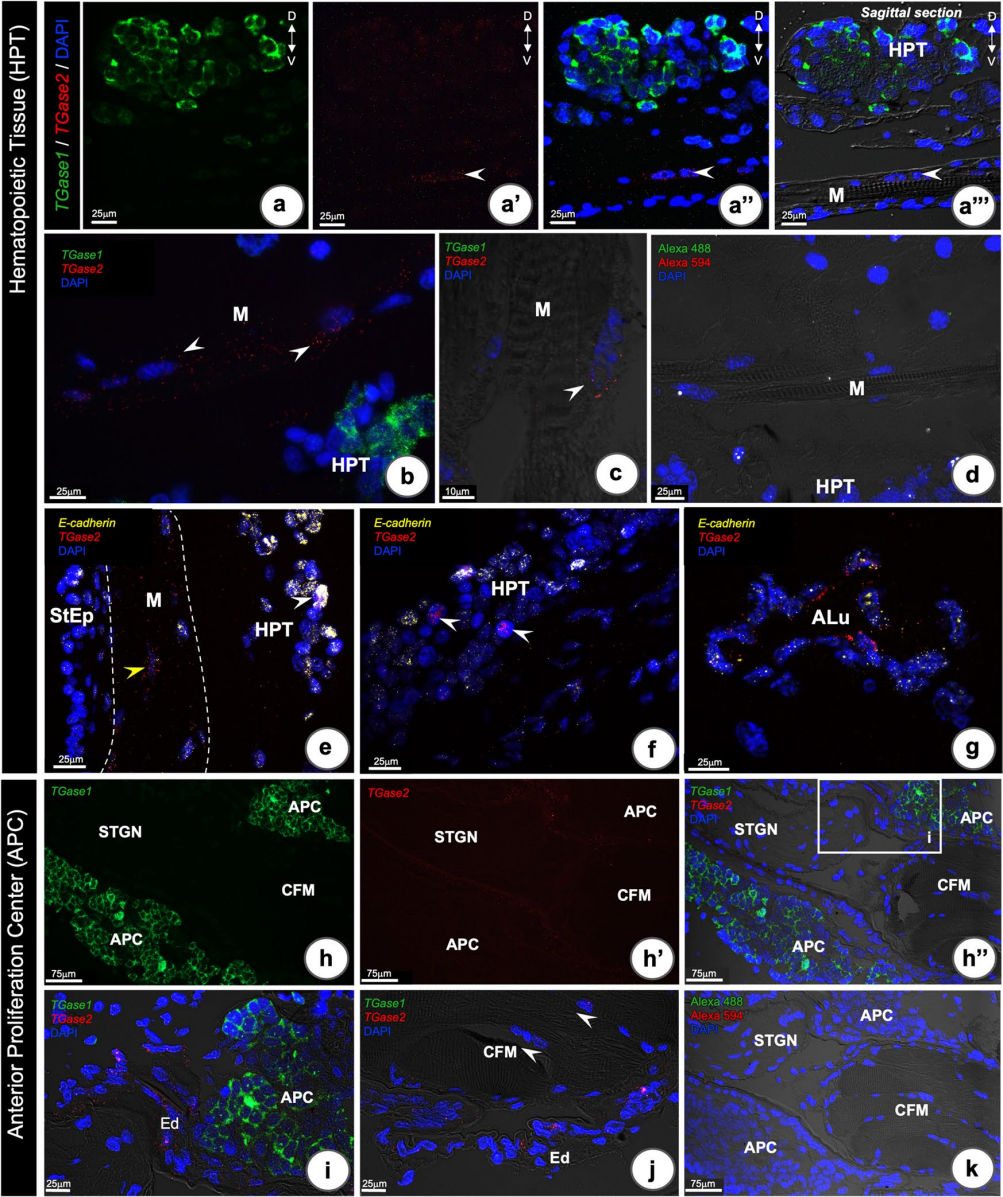
RNA-FISH detection of *TGase1*, *TGase2*, and *E-cadherin* transcripts in the HPT and APC of *P. leniusculus.* **a**–**a’’’** A sagittal section of HPT showing localization of *TGase1* (green, **a**), *TGase2* (red, **a’**), merged (**a’’**) and with over-laid differential interference contrast (DIC) (**a’’’**). **b**, **c** *TGase2* mRNA is located in cells associated with epithelial and muscular tissue underneath the HPT (arrowheads). **d** Negative control without probe. **e**–**g** Co-localization of *TGase2* and *E-cadherin* transcripts in HPT. **e** *TGase2* was detected in muscular tissue (yellow arrow head) and in a few HPT cells (white arrow heads in **e** and **f**) and clearly in the endothelial cells of the artery (**g**). E-cadherin was detected in some HPT cells (white arrow head in **e**). **h**–**j** Co-localization of *TGase1* and *TGase2* mRNA in the APC tissue. The *TGase1* transcript was observed in the APC cells (green, **h**; red, **h’;** merged, **h’’**), while the *TGase2* mRNA was found in the endothelial cells (Ed) lining the cerebral artery (**i**) and surrounding the CFM (arrow heads in **j**). **k** Negative control without probe. D, dorsal; V, ventral; CFM, Cor frontal muscle; StEp, stomach epithelium; M, muscular tissue; ALu, Arterial lumen; STGN, stomatogastric nerve

We have previously detected two different transcripts encoding transglutaminase enzymes (Junkunlo et al. 2020), and hence we decided to investigate in which cells these transcripts were localized. Simultaneous detection of *TGase1* and *TGase2* mRNA was performed using multiplex RNA-FISH in the HPT (Fig. 3a–g) and APC (Fig. 3h–k). While *TGase1* was expressed only in the HPT cells (Fig. 3a, a”, a”’, b), *TGase2* mRNA was detected in other tissues, a few cells in the muscular tissue underneath HPT (Fig. 3a’, a”, a”’, b, c, e), only a few HPT cells (Fig. 3e, f), and mainly in the endothelial cells (Fig. 3g). Moreover, we could show some *TGase2*-expressing cells in addition also expressed *E-cadherin* (Fig. 3e–g).

The localization of *TGase1* and *TGase2* mRNA in the APC exhibited the same expression pattern as in the HPT. *TGase1* was expressed in the APC cells (Fig. 3h, h”, i). while *TGase2* was detected in the endothelial cells of CA (Fig. 3h’, h”, i, j) as well as occasionally in the CFM (Fig. 3j).

These results show that the two transcripts, *TGase1* and *TGase2*, are expressed in different cell types, confirming our previous finding (Junkunlo et al. 2020) and may indicate distinct functions.

### Co-localization of TGase1 and Hml mRNA in the HPT, APC and hemocytes

**Fig. 4:**
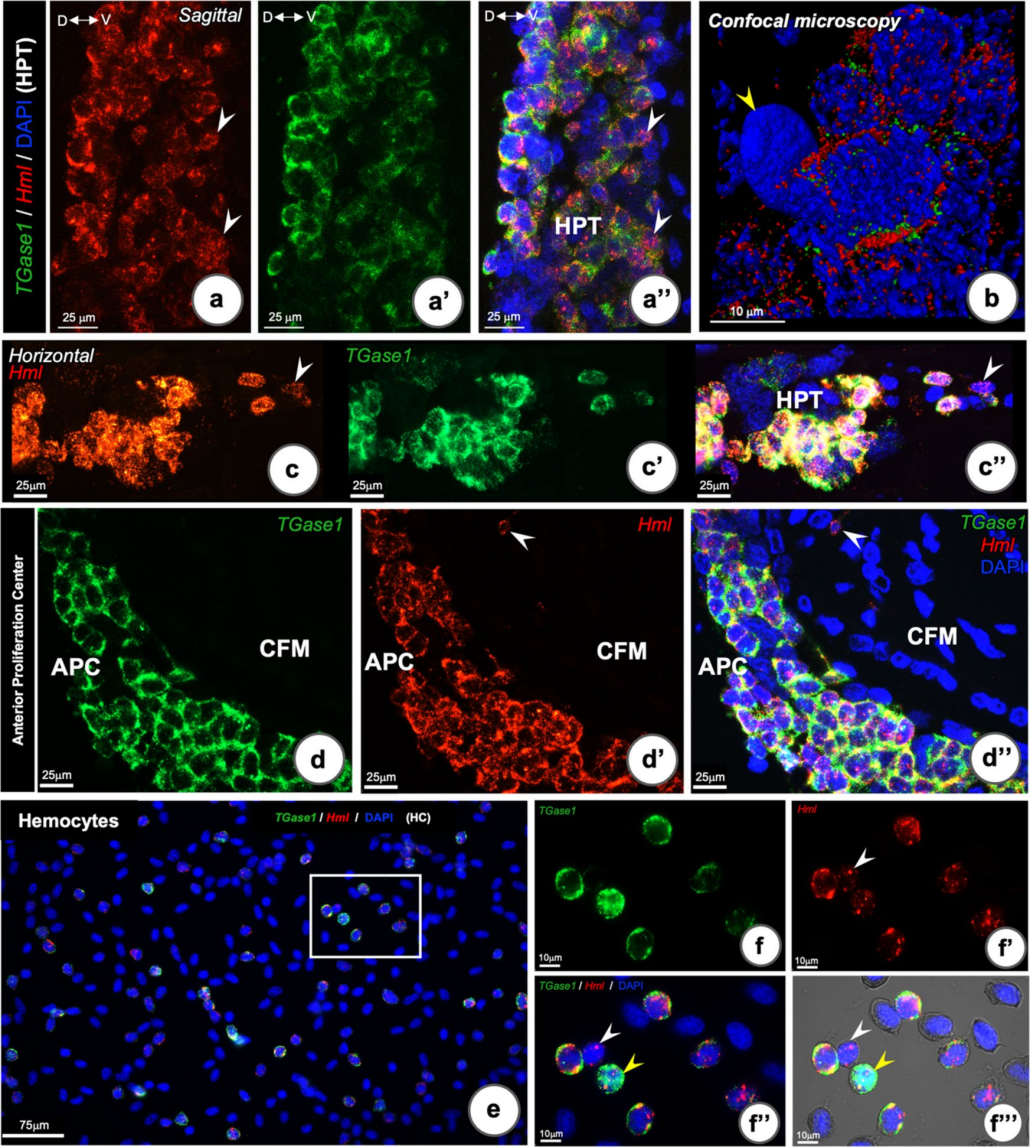
RNA-FISH co-localization of *TGase1 *and *Hml* transcripts in the HPT, APC, and hemocytes in *P. leniusculus,* respectively*.* **a** Sagittal section of HPT showing localization of *Hml* (red **a**) and *TGase1* (green **a’**) in the HPT tissues (merged **a’’**). **b** Confocal Z-stacking micrograph showing co-localization of *TGase1* and *Hml* in the same HPT cell, while the heterochromatic nuclear cell is absent of a positive signal (yellow arrow head). **c** Co-localization of *TGase1* and *Hml* transcripts detected in HPT cells, which have been released from the lobule. Most cells expressed both transcripts, whereas some cells expressed *Hml*, but not *TGase1*(arrow heads in **a** and **a**”, arrow head in **c** and **c**”). **d** Co-localization of *TGase1*and *Hml* transcripts in most APC tissue cells, while, *Hml* positive signal was detected in a few released hemocytes (arrowhead). **e**, **f** Co-localization of *TGase1 *and *Hml* transcripts in circulating hemocytes. **f**–**f’’’** Higher magnification of the square in (**e**). **f’’**, **f’’’** low *TGase1* signal (white arrowhead), medium co-transcription, and high *TGase1* signal (yellow arrowhead)

To further identify different cell types within the HPT, we chose to investigate the localization of mRNAs for *TGase 1* and hemolectin (*Hml*) in HPT simultaneously in both sagittal and horizontal sections. We selected these two transcripts since we earlier found a gradient in the expression of these two transcripts, with higher expression in cells in clusters with more mature cells by the use of single-cell RNA seq analysis (Söderhäll et al. 2022). The obtained results show that *TGase1*and *Hml* mRNA were most often present in the same HPT cells (Fig. 4a–c”), while in some cells, a more pronounced expression of *Hml* was evident and these cells were located close to the base of the lobules (Fig. 4a and a”) as well as in some already released cells (Fig. 4c and c”). Tissues without probe (non-probe) were used as controls for signal detection. As expected, no positive signal was detected in the non-probe-treated sections (Fig. S2a–a’’’). There was no difference in expression pattern between *TGase1*and *Hml* in the APC tissue (Fig. 4d and d”), except in some mature hemocytes associated with the CFM (Fig. 4d’ and d”). In hemocytes, there was a variation of cells which expressed both transcripts at different levels. All the cells that have *TGase1* mRNA also expressed *Hml*. In contrast, not all of the *Hml* mRNA-positive cells co-expressed *TGase1* mRNA. Thus, the *Hml*-positive hemocytes could be categorized into four main sub-populations based on their intensity of the simultaneous *TGase1* RNA-FISH signals (Fig. 4e–f”’); (1) low *TGase1* expressing hemocytes, (2) medium expressing *TGase1* hemocytes, (3) high *TGase1*-expressing hemocytes, and (4) no expression of *TGase* 1 (Fig. 4f–f’’’). We conclude that *Hml* and *TGase1* most often are expressed in the same cells, and this may indicate a function in regulating the extracellular matrix composition and strength.

### Pacifastin heavy chain and melanization inhibiting protein and their localization in HPT, APC and hemocytes

**Fig. 5:**
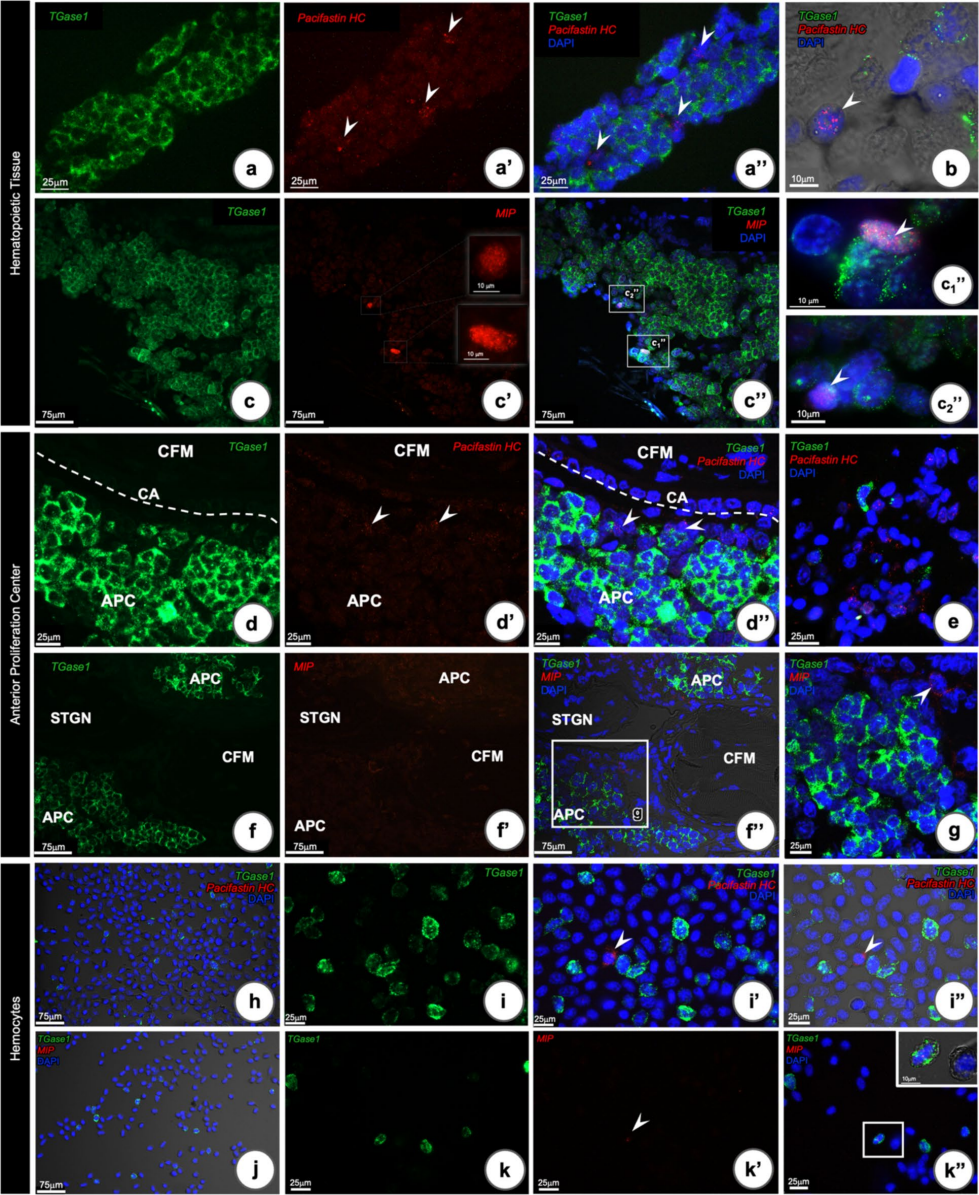
RNA-FISH localization of *TGase1*, *pacifastin heavy chain (HC)*, and *MIP* transcripts in the HPT, APC, and hemocytes of *P. leniusculus.* **a** *Pacifastin-HC* and *TGase1* co-localization in HPT, *Pacifastin-HC* positive cells (red **a’**) were observed in cells located close to the sinusoidal space of HPT, and they did not co-localize with the *TGase1* transcript (**a**) in the same cells (arrow heads, merged **a’’**), **b** higher magnification showing a *Pacifastin-HC* expressing cell. **c** *MIP* and *TGase1* co-localization in HPT; *MIP* positive cells were observed only in a few cells located in the periphery of the HPT **c’**, **c’’**. **c**_**1**_**″** and **c**_**2**_**″** Higher magnification of squares from **c”**, showing co-localization with *TGase1* transcript. APC: **d** *Pacifastin-HC* and *TGase1* co-localization in APC, *Pacifastin-HC* positive signal was found in the cells located close to the endothelial lining of the cerebral artery (arrow heads in **d’** and **d”**), **e** *Pacifastin-HC* transcripts were also detected in the periphery of APC associated with connective tissue. **f** *MIP* and *TGase1* co-localization in APC, *MIP* positive signals were observed in cells located in the space between APC lobules and the endothelial layer of cerebral artery (*TGase1*green **f**, *MIP* red **f’**, merged **f’’**), **g** higher magnification in squares from **f’’** showing MIP expressing cell (arrow head in). Hemocytes: **h** Circulating hemocytes labeled with green labeled probe for *TGase1* and red labeled probe for *Pacifastin-HC*. **i** Occasional hemocytes were found to express *Pacifastin-HC* (arrow head in **i’** and **i’’**); **j** circulating hemocytes labeled with green labeled probe for *TGase1* and red labeled probe for *MIP*. **k**, **k”** *TGase1* transcripts were observed in few cells, and *MIP* transcripts were detected only occasionally and then co-localized with *TGase1* (arrowhead in **k’** and insert of **k”**)

Pacifastin heavy chain (pacifastin-HC) positive signals were detected in some HPT cells, and as expected from our previous study (Söderhäll et al. 2022), it did not co-localize with *TGase1*- or *Hml*-positive cells (Fig. 5a’–b). In contrast, there was co-localization of *MIP* mRNA signals in a few of the *TGase1*-positive cells (Fig. 5c”, c_1_″, c_2_″). The pattern of *pacifastin-HC* mRNA localization in the APC was identical to that occurring in the HPT cells. These cells were localized close to the CA (Fig. 5d–d”), and were present in the periphery of the APC (Fig. 5e). Only occasionally a few hemocytes could be found expressing *pacifastin-HC* mRNA (Fig. 5h, 5i’, and i”). In contrast, *MIP* mRNA was not observed to be co-localized with *TGase1*-expressing APC cells as it sometimes was in HPT. Only a few *MIP* positive cells were detected in the hemal space between APC and CA-endothelium, whereas no positive *MIP* signals could be detected in the APC (Fig. 5f’–g). In hemocytes, only very few cells with low *MIP* expression were found, and these hemocytes were also *TGase1* positive (Fig. 5j–k”). Thus, *MIP* and *pacifastin-HC* are expressed in very few cells and almost exclusively in HPT and APC, highlighting the need for further detailed studies to understand their functions.

### Endoglucanase (cenB) and PDGF/VEGF-like type 3 mRNA localization in HPT, APC and hemocytes

In our recent single-cell RNA sequencing study (Söderhäll et al. 2022), we did identify some previously unknown transcripts, which were localized in two separate hemocyte clusters, namely an endoglucanase (*cenB*) and a PDGF/VEGF-like factor (comp33904_c0, GenBank accession GBYW01023797) now named as *PVF3*. Therefore, we decided to look in more detail in which cells these transcripts could be detected.

**Fig. 6:**
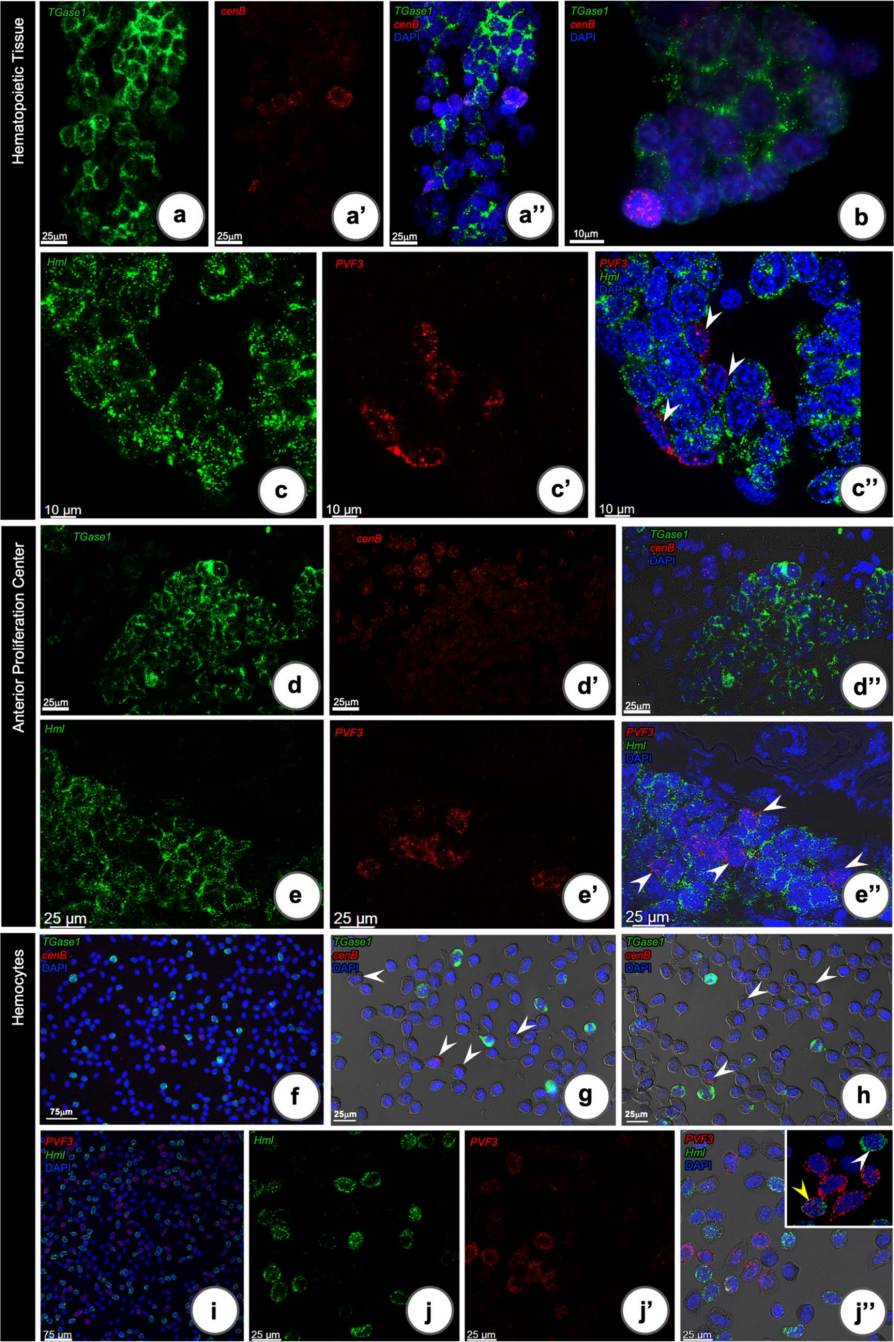
RNA-FISH localization of *TGase1*, *Hml*, *cenB*, and *PVF3* transcripts in the HPT, APC, and hemocytes of *P. leniusculus.* In the HPT (**a**–**c**): **a**, **a”** *cenB* positive cells were observed in a few cells in the HPT lobules, but these cells were different from the *TGase1* positive cells. **b** High magnification of *cenB* positive cells. **c**, **c”** *PVF3* positive cells were observed in the HPT lobules in cells that were different from the *Hml* positive cells in the HPT lobule. In the APC (**d**, **e**): **d**, **d”** *cenB* positive cells were observed in the peripheral area of APC in cells without presence of *TGase1* transcripts. **e**, **e”** *PVF3* positive cells were observed in cells without any detectable *Hml* transcripts (arrow heads). In hemocytes (**f**–**j**): **f** Localization of *cenB* and *TGase1* transcripts in hemocytes. **g**, **h** *cenB* transcripts (arrowheads) are localized in other cells than *TGase1*. **i** Confocal microscopy showing the localization of *Hml* and *PVF3* transcripts in hemocytes. **j**, **j’’** Four cell types were observed; most *PVF3* positive cells did not express *Hml* and a few cells expressed *Hml* and *PVF3* (yellow arrowhead), while most *Hml* positive cells (white arrowhead) did not express *PVF3*, some cells did not express neither *Hml* nor *PVF3* (only DAPI-stained cell)

Expression of *cenB* mRNA was found in both HPT and APC as well as in hemocytes. In the HPT the mRNA of *cenB* was found in cells without *TGase1* transcripts and inside the HPT lobules (Fig. 6a, a’, a”). Most *cenB* positive cells were located close to the luminal area, or in contact with the hemal sinus, indicating a more mature stage (Fig. 6a” and b). This expression pattern was also observed in the APC (Fig. 6d–d’’). In hemocytes, the expression of *cenB* mRNA was found in cells lacking the *TGase1* transcript (Fig. 6f, part of f magnified in g, and h).

The expression pattern of the *PVF3* gene detected by RNA-FISH was clearly distinguishable from *Hml-*positive cells in the HPT (Fig. 6c, 6c’, and c”). Notably, all positive *PVF3* cells were located close to the HPT lumen or at the interface within the hemal space (Fig. 6c”). No positive signal was observed from the negative control which are the tissues without probe treatments (non-probe) (Fig. S2b–b”’). Similarly, no co-localization of *PVF3* and *Hml* mRNA was detected in the same APC cells. Some *PVF3* positive cells were scattered around in the APC lobules, whereas a few cells were located close to the hemal sinus (Fig. 6e”). Also, in hemocytes *PVF3* and *Hml* mRNA were detected in different cell types (Fig. 6i and j–j”). We could find at least four subpopulations of hemocytes categorized based on the intensity of the RNA-FISH signals (Fig. 6j”, insert, and magnified in Fig. S3a). The first cell type had no visible mRNA expression of *PVF3* or *Hml.* The second cell type had high *PVF3* signal, without any *Hml.* The third cell type, which were few, contained both *Hml* and *PVF3* mRNA, while most *Hml* positive cells did not express *PVF3* and was classified as belonging to the fourth type (Fig. S3).

Taken together, these results indicate that *cenB* and *PVF3* expression suggest that these are more developed precursor cells prepared for release into the circulation, with functions distinct from those of *Hml*- or *TGase1*-expressing cells.

### Proliferating cell detection by BrdU incorporation in the HPT and APC and co-localization with TGase1 or Pacifastin-HC protein

**Fig. 7:**
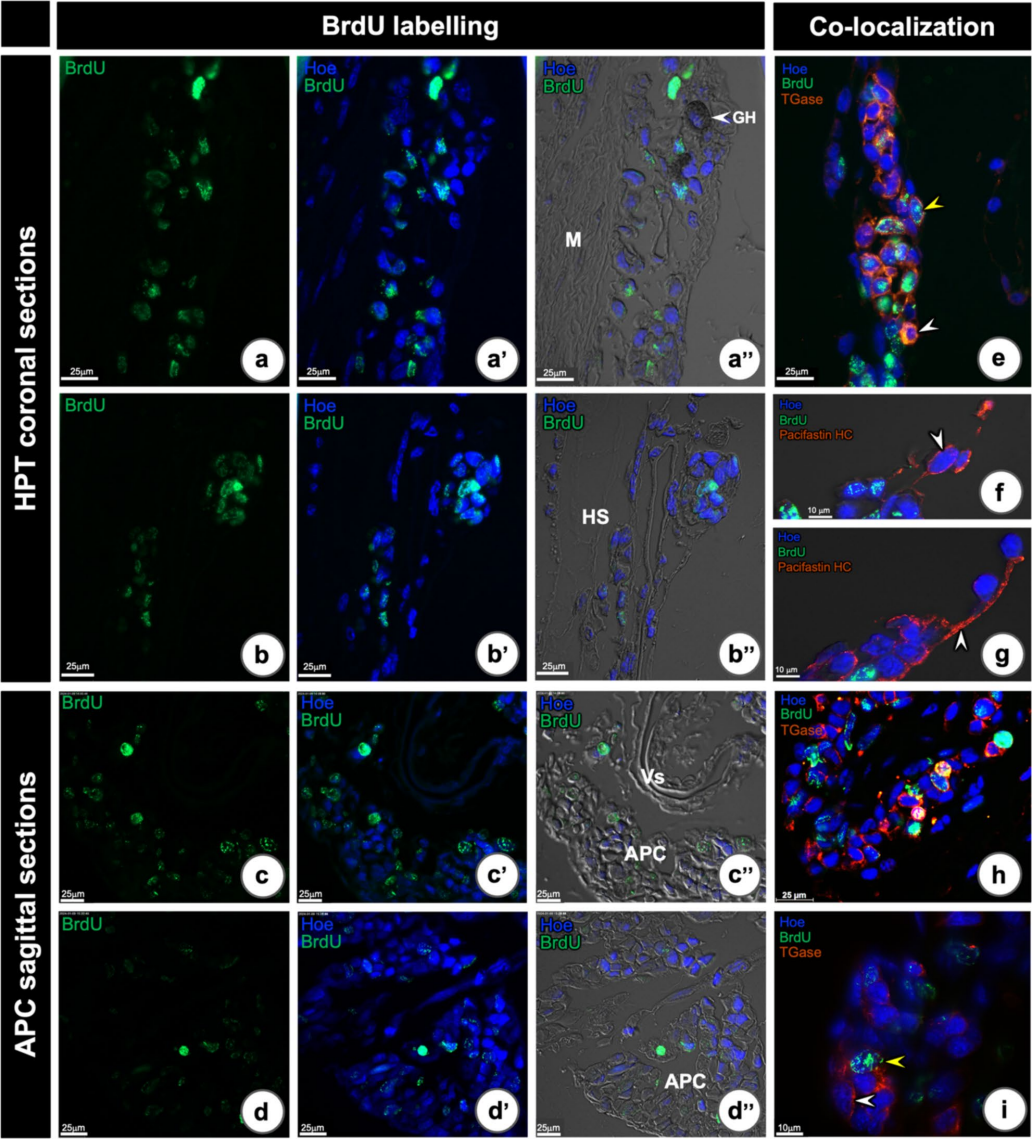
Proliferating cells detected by BrdU incorporation in the HPT and APC, and their co-localization with TGase1 and Pacifastin-HC protein in HPT and APC, respectively. HPT:** a**, **a”** and **b**, **b”** BrdU positive cells were observed irregularly dispersed throughout the HPT (**a**, **a”**) and inside HPT lobules (**b**, **b”**). APC: **c**–**c”** and **d**–**d”** BrdU positive signal was detected in a similar pattern as in HPT and in the APC cells. **e** Co-localization of the BrdU and TGase proteins in the HPT tissue. Three main cell types were found: (1) only BrdU positive cells without TGase protein, (2) cells with BrdU and TGase positive signals (yellow arrowhead), and (3) only TGase positive signal without BrdU incorporated nuclei (white arrow). **f**, **g** Co-localization of BrdU and pacifastin-HC protein in the HPT tissues. Pacifastin-HC protein was found surrounding the periphery of the lobules (**f**, arrowhead) and associated with the thin membrane covering HPT lobules (**g**, arrowhead). APC: **h** and **i** Co-localization of BrdU and TGase proteins in the APC showed a similar pattern as in the HPT [**i** only TGase positive cells (white arrowhead) and colocalization of BrdU and TGase positive signals (yellow arrow head)]. GH, granular hemocyte; Vs, vessel

We then performed BrdU incorporation in order to localize proliferating cells in the HPT and APC, together with a simultaneous detection of the TGase1 or pacifastin-HC protein. As we have reported earlier (Noonin et al. 2012), BrdU positive cells were dispersed throughout the HPT (Fig. 7a–a” and b–b”) and in the APC (Fig. 7c–c” and d–d”). In the HPT, TGase1 immunoreactivity was abundant in the cytoplasm of cells which were not BrdU positive (Fig. 7e, white arrowhead), and with intermediate signal of TGase1 protein in the cells which were BrdU positive (Fig. 7e, yellow arrowhead). However, several HPT cells were TGase1 positive but without any signs of BrdU labeling.

Pacifastin-HC protein was not present in the BrdU-labeled cells and was found in the cytoplasm of a few cells located close to the sinus (Fig. 7f). However, most of the pacifastin-HC protein was found to be located on the outside of the cells, associated with a connective membrane packing the cells at the apical surface of the tissue (Fig. 7g). TGase1 immunoreactivity was also detected in the APC (Fig. 7h), and the TGase1 protein was found in a similar pattern as that described in the HPT (Fig. 7i), whereas no pacifastin-HC immunoreactivity was detected in the APC (data not shown). Negative controls without anti-BrdU and anti-pacifastin-HC antibodies incubation were conducted and, as expected, showed no signs of positive signals when observed under the same fluorescent intensity (Fig. S4a–a””).

All together the localization of S-phase cells by BrdU incorporation showed that these cells are scattered around HPT and not concentrated in specific areas, and in addition cells expressing *TGase1* are cells with proliferating capacity.

### Subpopulations of mature hemocytes identified by co-localization of RNA-FISH and immunofluorescence techniques

**Fig. 8:**
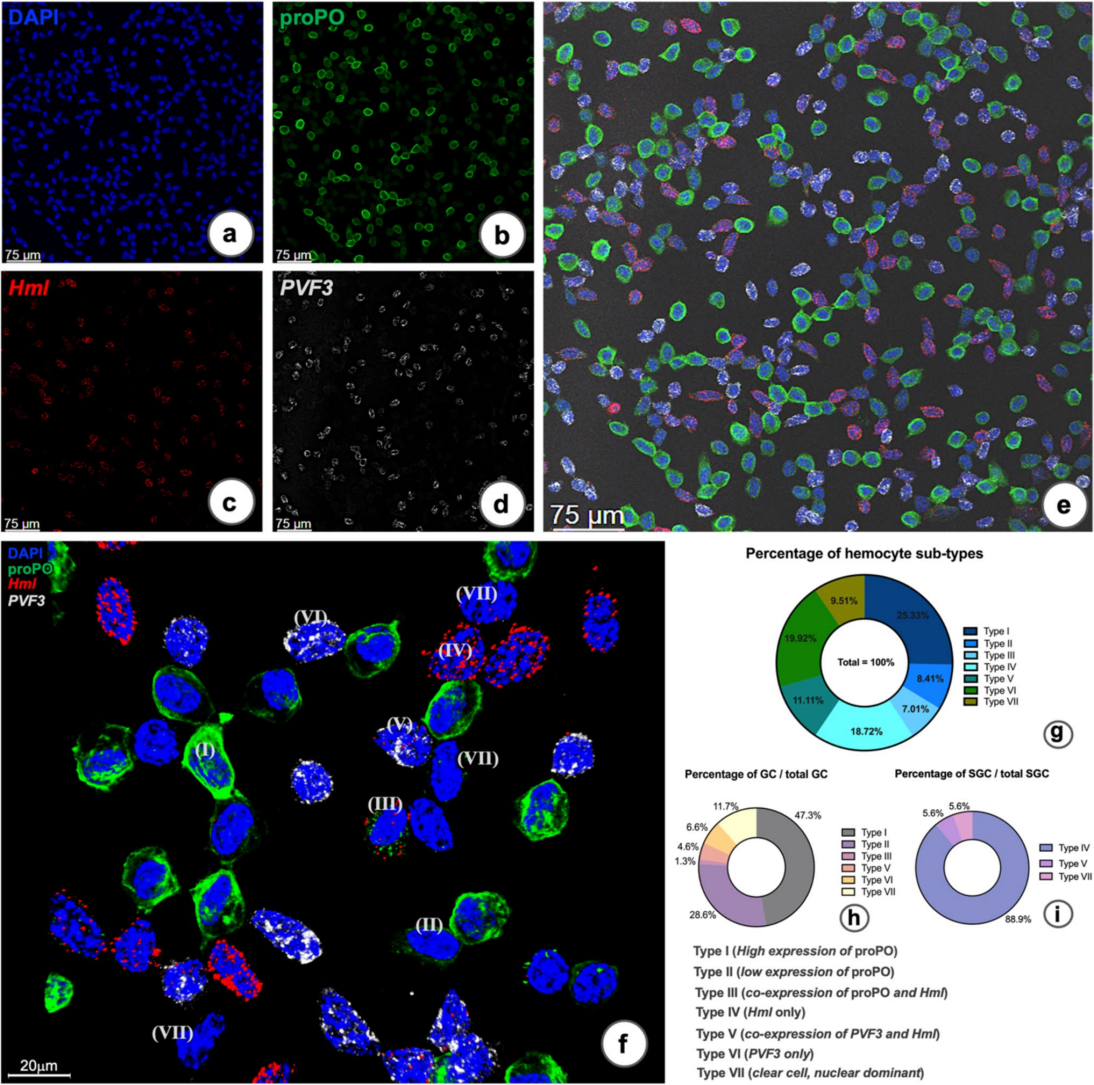
Combination of RNA-FISH (*Hml* and *PVF3* transcripts) and immunofluorescence with an antibody specific to the proPO N-terminal in hemocytes of *P. leniusculus.* **a**–**f** Confocal fluorescence microscopic pictures of DAPI nuclear staining (**a**), proPO protein positive signal (**b**), RNA-FISH signals of *Hml* (**c**), and *PVF3* (**d**) transcripts, respectively, in the hemocytes (a-d are merged in **e**). **f** Z-stacking 3-D rendered pictures from confocal microscopy of hemocytes and they were stained as above (merged from Fig. S5a–a’’’). The cells could be classified into seven main subtypes based on the three-positive signals: (I) High proPO expressing hemocytes, (II) low proPO expressing hemocytes, (III) low proPO co-expressing cells with *Hml* transcript, (IV) only *Hml* expressing cells, (V) low expression of *Hml,* high expression of *PVF3*, (VI) only *PVF3* expressing cells, and (VII) clear cell or nuclear dominant hemocyte, very low or absent expression of other genes and protein. **g** Percentage of all seven hemocyte subtypes per total hemocytes. **h** Percentage of the granulocyte subtypes by fluorescent label classification per total granulocyte. **i** Percentage of the semigranular subtypes by fluorescent-labeled classification per total semigranular cells. Similar results were obtained from different animals

In order to find the expression pattern of some of the transcripts in circulating hemocytes, we performed RNA-FISH in combination with staining for the prophenoloxidase (proPO) protein. We then used an antibody against the N-terminal part of proPO to test for the presence of uncleaved, not activated proPO protein (Jearaphunt et al. 2014). By the combination of RNA-FISH for *Hml* (Fig. 8c and Fig. S5a”) and *PVF3* (Fig. 8d and Fig. S5a”’), and immunofluorescence against the proPO N-terminal (Fig. 8b and S5a’) in hemocytes followed by counterstaining the nuclei with DAPI (Figs. 8a and S5a), it was possible to classify these cells into seven subpopulations based on their expression patterns (Fig. 8e which is a merged version of Fig. 8a–d, and f which is merges Fig. S5a–a’’’)*.* The hemocytes intensely stained for the proPO protein were classified to be type I. The hemocytes with pale intensity of proPO immunoreactivity was classified to be type II. Hemocytes with a low amount of proPO protein together with *Hml* mRNA was type III. Only *Hml* expressing cells were categorized to be of type IV. Low expression of *Hml* and high expression of *PVF3* in hemocyte were classified as type V and only *PVF3*-expressing hemocytes as VI, and finally hemocytes expressing neither *Hml*, or *PVF3* mRNA, nor proPO protein were classified as type VII (Fig. 8f). The percentage of each sub-population in this example is shown in Fig. 8g. The results showed that the hemocyte types detected were the proPO positive cells type I (25.3%) and II (8.4%), respectively (Fig. 8g). Hemocyte type IV, which were only *Hml* positive (18.7%), were all of the semigranular morphotype as earlier shown (Söderhäll et al. 2022). However, type VI, the *PVF3* positive hemocytes (19.9%), were largely of the granular cell type, but without any expression of the proPO protein. Some of the *Hml* positive cells did express proPO (7%) and a few other *Hml* positive cells expressed PVF3 (11.1%). About 9.5% did not express any of these tested transcripts or proPO protein (VII). Note that the *PVF3* transcript was never found in combination with proPO protein. The hemocyte types III, V, and VII were 7.0%, 11.1%, and 9.5% of the total populations, respectively (Fig. 8g). If their proportions are taken into consideration, all type III hemocytes belong to granular cells 1.3% (Fig. 8h), while types V and VII were a mixture of granular hemocyte (4.6% and 11.7%, respectively) and semigranular hemocyte (5.6% and 5.6%, respectively) (Fig. 8h and i) characteristics.

In conclusion, based on our results exemplified in Fig. 8, we find that the cell population in *P. leniusculus* is highly diverse, encompassing a greater variety of hemocyte types than can be classified solely by morphological criteria.

## Discussion

The hematopoietic tissue (HPT) and anterior proliferation center (APC) are well established as the hematopoietic organ in freshwater crayfish, *P. leniusculus* (Söderhäll and Söderhäll 2022). The HPT is located on top of the crayfish stomach and contains layers of hematopoietic ovoid-shaped lobules inside a thin network of connective tissue. In an effort to determine how maturing hemocytes enter into the circulation, we observed HPT sections after injection of India ink. As shown by Chaves da Silva et al. (2013), the anterior median artery (AMA) is located between the right and left HPT sheath, directly below the carapace without any HPT tissue surrounding the top area of this artery. The location of crayfish HPT closely to the AMA is similar to the corresponding structures in insects, e.g., lymph gland and dorsal artery in *Drosophila* larvae reviewed by Grigorian and Hartenstein (2013). The AMA does not seem to be in direct contact with the HPT, but it is enclosed in a layer of hemal sinus like the artery in the lacunar of venae system. This hemal sinus connects continuously into the hemal space beneath the HPT. The ink injection showed that there is no leakage of ink between the AMA and the HPT, and therefore it is not likely that HPT cells are released into this artery but instead enter the hemocoel through the hemal sinuses, as suggested by Chaves da Silva et al. (2013), and as is shown in insects (Hoffmann et al. 1979; Grigorian et al. 2011; Koranteng et al. 2022). Localization of the APC as part of the hematopoietic organs in association with the cor frontal structure was confirmed in this study. The existence of cor frontale muscles (CFM) linked to the APC in crustaceans has previously been reported and described to function as an extra heart (Chaves Da Silva et al. 2013; Pudgerd et al. 2019). Our study confirms that CFM strands are present in the lumen of the terminal segment of the AMA, formerly called the cerebral artery (CA). We also found the stomatogastric ganglion (STG) and its nerve in the same position as previously reported (Steinacker 1978; Huckstorf and Wirkner 2011; Machon et al. 2019). However, we did not find released APC cells (maturing hemocytes) inside of the arteries in connection with the cor frontal, but instead release of cells is more likely into the hemal sinus (APC canals), and whether there is any connection for APC cells via the cor frontal-associated arteries to the brain area is still not completely clear.

The lobular organization of the HPT in *P. leniusculus* differs markedly from that in the giant freshwater prawn., *M. rosenbergii* (Pudgerd et al. 2019). In *M. rosenbergii* the HPT lobules were packed close to the central hemal sinus and could be classified into three different zones from the proximal to distal of a lobule. Based on H&E staining and BrdU incorporation studies, we found that the lobules in *P. leniusculus* were more irregularly organized in the tissue and in each individual lobule, cells in different stages of differentiation were present. This irregular organization was also confirmed by RNA-FISH detection of *Hml* and *TGase1*, the transcript of which we previously showed were increasing during differentiation (Söderhäll et al. 2022). The expression of these transcripts was spread in all lobules at different levels, while there were cells in the lobules which were lacking *Hml* and *TGase1* expression and hence may be less-differentiated stem cells. However, this has to be confirmed after finding specific markers for these cells (Table 2).

**Table 2:** A summary of the levels of gene and protein expression in tissues and hemocytes

	Levels of positive signals and localization								
Tissues	*TGase1*	*TGase2*	*Hml*	*Pacifastin-HC*	*MIP*	*cenB*	*PVF3*	proPO^a^	Remarks
HPT	** + + + + + **	** + **	** + + + + + **	** + **	** + **	** + **	** + + + **	N/A	*TGase2* detected in subHPT muscles and endothelial cell
APC	** + + + + + **	** + **	** + + + + + **	** + **	** + **	** + + **	** + + + **	N/A	*TGase2* detected in CFM^b^ and endothelial cell
Hemocytes	** + + + + **	** + + **	** + + + + **	**(0/ +)**	**(0/ +)**	** + **	** + + + **	** + + + + + **	(0/ +) few cells were detected

+ , very low expression; 0/ + , very few expressing cells; + + , low expression; + + + , moderate expression; + + + + , high expression; + + + + + , abundant expression; N/A, not applicable

^a^proPO protein; ^b^CFM; Cor frontal muscle

Cells within the APC which is surrounding the cor frontal were less organized into lobules compared to HPT cells as earlier reported in the crayfish *P. clarkii* (Chaves Da Silva et al. 2013). However, small-sized lobules or rosette-like structures could be observed in posterior and peripheral parts of the APC. Compared to HPT cells, we found that cells with heterochromatic nuclei were much more abundant in the APC, indicating less-differentiated cells. However, the spatial expression of *Hml* and *TGase1* transcripts in APC showed a similar pattern to that in HPT. This may indicate that some mature hemocytes are released from the APC in addition to the HPT. Immunostaining using TGase1 antibody together with BrdU incorporation similarly showed that very high TGase1 protein levels were not found together with S-phase cells. Both *TGase1* and *Hml* mRNA were found in hemocytes, but only in a small number of cells in accordance with our previous single-cell RNA sequencing analysis (Söderhäll et al. 2022).

We interpret these results as that both TGase1 and Hml have important functions in HPT and APC, and that circulating hemocytes expressing these transcripts are relatively recently released from the tissues and later differentiate into different types of functional hemocytes. As previously shown, TGase1 enzyme activity is important for crosslinking extracellular matrix proteins, keeping the stem cells in an undifferentiated stage (Söderhäll 2016). However, the function of Hml in hematopoiesis in crustaceans is not known, which is in contrast to several reports on Hml expression in plasmatocyte precursors in the lymph gland of *Drosophila* (Spratford et al. 2021; Kharrat et al. 2022). Hemolectin in *Drosophila* participates in the coagulation reaction (Lesch et al. 2007) and its absence results in bleeding defects during wounding (Goto et al. 2003). In crayfish coagulation is achieved by TGase1 crosslinking of a clotting protein (Hall et al. 1999; Huang et al. 2004), and moreover, this clotting protein is part of the extracellular matrix in the HPT. A similar function in the ECM for *Hml* as a large secreted protein would be possible but still needs to be shown.

While TGase1 is abundant in HPT and APC, TGase2 was mainly expressed in other cell types and most obvious in the epithelial or endothelial cells where it was colocalized with E-cadherin, but also in a few muscle-associated cells and very few HPT cells (Table 2). This indicates that TGase2 and TGase1 have very different functions, which needs further investigation (Junkunlo et al. 2020). An interesting observation was that E-cadherin was clearly expressed in some HPT cells, which may indicate specific precursor types of cells, as is the case in mice where E-cadherin was shown to be highly expressed in basophil and mast cell precursors (Wanet et al. 2021).

To follow up our previous single-cell RNA-sequencing study, we located some of the transcripts that were particularly interesting, namely pacifastin-HC, MIP, cenB, and PVF3. These transcripts were detected in separate clusters, different from Hml, TGase1, and well-known immune genes such as proPO, crustins, and other antimicrobial proteins (Söderhäll et al. 2022). Our present study confirms that *pacifastin-HC* was exclusively expressed in HPT and APC cells and in cells not expressing *Hml* or *TGase1*. It was more abundant in APC, and in the cells located close to the endothelial lining of the cerebral artery and in the periphery of APC associated with connective tissue. Pacifastin-HC protein was detected surrounding the lobules and also associated with the membrane surrounding the tissue. There may be two putative functions of pacifastin-HC: first to transport excess iron from cells undergoing mitophagy or apoptosis, and secondly, its association with the pacifastin light chain to inhibit proteases (Liang et al. 1997).

The melanization-inhibiting protein MIP was also expressed not only in both APC in cells devoid of *Hml* and *TGase1* expression but also in a few cells in HPT together with low *TGase1* expression. As MIP inhibits the melanization reaction carried out by phenoloxidase (Söderhäll et al. 2009), its activity may be of high importance in the APC and HPT to avoid melanin formation and phenol derivatives inside the tissues which can be toxic to the cells.

Two previously unknown *P. leniusculus* transcripts were localized to specific hemocyte types in this study. First, a putative endoglucanase named cenB was expressed in the peripheral areas of the APC and in specific cells without *Hml* or *TGase1* expression in outer parts of HPT lobules, and also in some hemocytes (Table 2). Crustacean endoglucanase (cenB) was first characterized in *C. quadricarinatus* (Byrne et al. 1999) and was present in F-cells of the hepatopancreases. It was proposed to function in cellulose hydrolysis in the herbivorous land crab (*Gecarcoidea natalis*) and crayfish (*Cherax destructor*) (Allardyce and Linton 2008). Cells expressing *cenB* may be a specific cell type, and the function of which needs to be determined in more detail. We also detected specific cells expressing a previously unknown PDGF-like factor named as PVF3. Of note is that we have previously characterized a PDGF/VEGF receptor-like protein with a putative role in hematopoiesis (Junkunlo et al. 2017). These *PVF3*-expressing cells were fairly abundant in the hemolymph and were also detected in the APC and HPT at the edges of the lobules, indicating more mature precursor cells. We consider *PVF3*-expressing cells as a specific cell type mainly of granular morphology, but without any proPO expression (Table 2), which is an interesting observation since all granular cells are considered to contain proPO. Moreover, the co-localization of *Hml* and *PVF3* mRNA with proPO protein clearly showed that there are more than two different cell types or lineages depending on its definition. This is detailed in Table 3 below.

**Table 3:** A table summarizing the levels of gene (*Hml* and *PVF3*) and protein (proPO) expression in hemocyte types

	Levels of positive expression in different hemocytes		
Types of HC	*Hml*	*PVF3*	proPO^a^
HC type I	**0**	**0**	** + + + + + **
HC type II	**0**	**0**	** + + **
HC type III	** + **	**0**	** + **
HC type IV	** + + + + + **	**0**	**0**
HC type V	** + + **	** + + + **	**0**
HC type VI	**0**	** + + + + **	**0**
HC type VII	**(0/ +)**	**(0/ +)**	**(0/ +)**

^a^ proPO protein, (0/ +) a few dots of signal/absent

## Conclusion

This study confirms some of the recent findings of a single-cell RNA sequencing of APC, HPT, and hemocytes from *P. leniusculus* (Söderhäll et al. 2022). We also showed that HPT lobules contain a diversity of cell populations, including hetero- and euchromatic nuclei, expressing *Hml* and *TGase1* in the majority of cells, while cells expressing *pacifastin-HC*, *PVF3*, or *cenB* were expressed in only few cell types. The hemal sinusoid or sinus surrounds the HPT, in which hemocytes are released. The APC has a more unclear lobular formation; the heterochromatic and euchromatic nucleated cells are tightly packed and closely associated with the cerebral-AMA arterial system. Our RNA-FISH results also show clearly different localization and putative different functions of the two transglutaminases TGase1 and TGase2, and moreover open for new investigations about E-cadherin as a possible marker for specific cell types. This study shows that the immune function of hemocytes in crustaceans is more complex and diversified than previously considered.

## Supplementary Information

Below is the link to the electronic supplementary material.

Supplementary file1 (DOCX 15.2 MB)

## Data Availability

No datasets were generated or analysed during the current study.
