# Supplementary material for: The hematopoietic tissue of the freshwater crayfish, Pacifastacus leniusculus: organization and expression analysis
Source: Cell Tissue Res. 2025 Jan 4;399(3):303–22. doi: 10.1007/s00441-024-03943-1 (PMC11870977; doi:10.1007/s00441-024-03943-1)
Supplement: Supplementary file 1 — Supplementary file1 (DOCX 15.2 MB) [file 441_2024_3943_MOESM1_ESM.docx]

**The hematopoietic tissue of the freshwater crayfish, *Pacifastacus leniusculus* ; organization and expression analysis.**

Thanapong Kruangkum^1,2,3^, Kenneth Söderhäll^1^, Irene Söderhäll^1^

^1^ *Department of Organismal Biology, Uppsala University, Norbyvägen 18A, 75236 Uppsala*

Present address:^2^ *Department of Anatomy, Faculty of Science, Mahidol University, Bangkok, Thailand,* and

^3^*Center of Excellence for Shrimp Molecular Biology and Biotechnology (CENTEX Shrimp), Faculty of Science, Mahidol University, Bangkok, Thailand*

Correspondence: Irene.Soderhall@ebc.uu.se

**Cell and Tissue Research**

**Supplementary material**

**Supplementary Table S1**. Custom branched probes for use with ViewRNA in situ hybridization assays (Invitrogen) were designed and provided by ThermoFisher Scientific according to the sequences below.

| **Transcript** | **Accession number** | **Fluorophore** |
| --- | --- | --- |
| cenB | GBYW01004615 | Alexa Fluor 594 |
| E-cadherin | GBYW01051896.1 | Alexa Fluor 594 |
| Hemolectin | GBYW01036052 | Alexa Fluor 594 |
| MIP | EU308499 | Alexa Fluor 594 |
| Pacifastin HC | U81824 | Alexa Fluor 594 |
| PVF3 | GBYW01023797 | Alexa Fluor 647 |
| TGase1 | AF336805 | Alexa Fluor 488 |
| TGase2 | MN913337 | Alexa Fluor 647 |

**Supplementary Fig. S1**

**
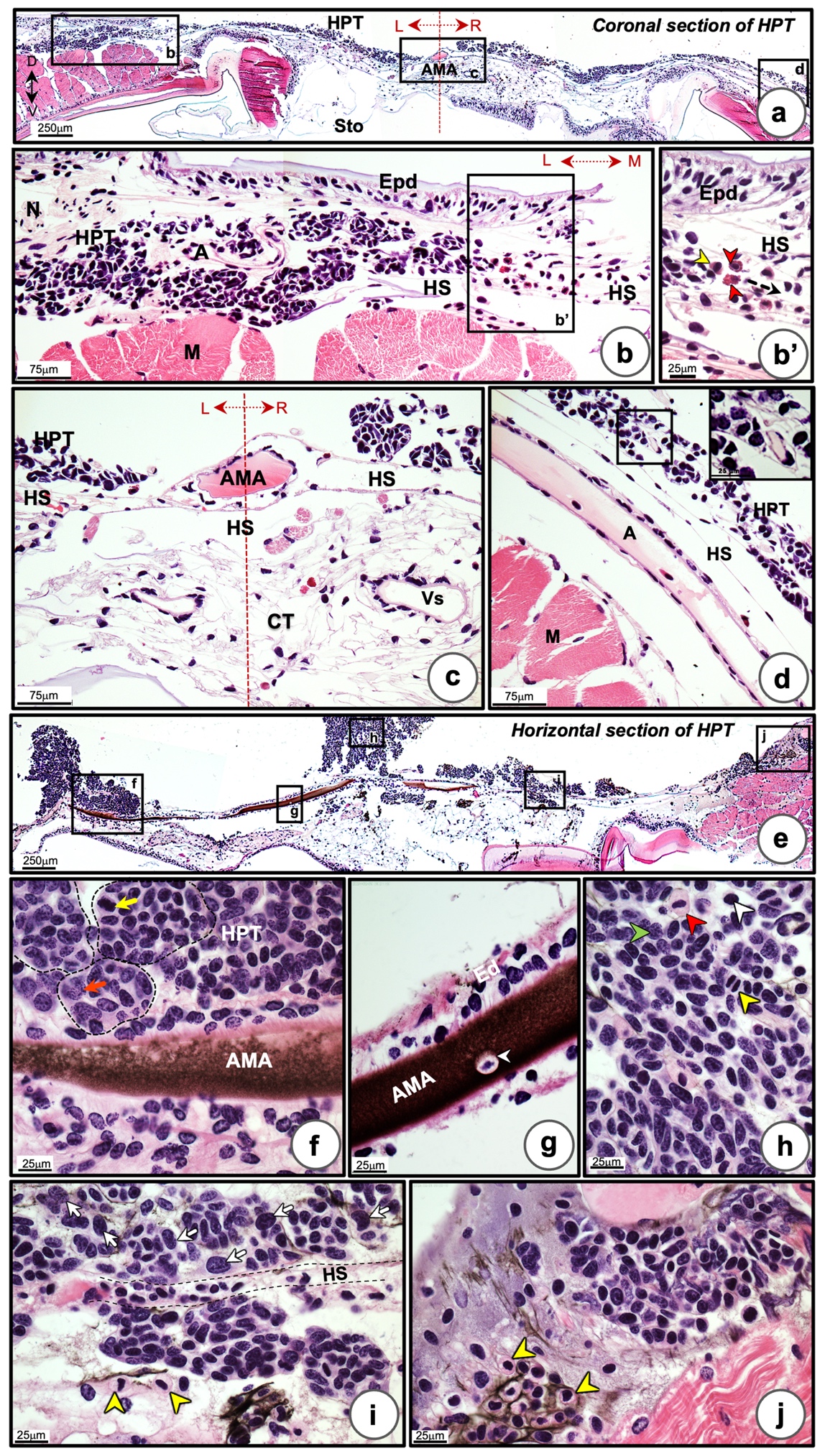
**

**Fig. S1** H&E staining of coronal and horizontal sections of the HPT. **a** A low magnification of the coronal section of HPT passing through the posterior edge of the stomach (Sto). Red dotted lines indicate the mid line. **b** The lateral margin of HPT is in close association with the epidermis (Epd) where the HPT is found in the sub-epidermal layer. Compact organization of HPT lobules dominates at the lateral margin and the area close to the mid line (the median artery lining as shown in **a**). In contrast, the HPT lobules are more loosely organized at the intermediate area in association with the hemal sinus (HS). **b’** Mature hemocytes; putative granular (red-arrow heads) and semi-granular hemocytes (yellow-arrow head) were found close to the HPT lobules and HS. **c** At the median site of section, the anterior median artery (AMA) is enveloped with a thin membrane of HS endothelium (called “periarterial sinus”) located below to the HPT lobules. **d** Small arterial vessels (capillary) are associated with the HPT lobules, while, the large arterial vessel (A) at the lateral side of body is found beneath of the HS. **e** A horizontal-low magnified section of HPT with an ink-signal in the AMA. **f** The HPT lobule is associated with the endothelial cells of the AMA; dashed lines represent lobules in the HPT. Dividing cell were found in some lobules (metaphase cell, yellow arrow; prophase cell, red arrow). **g** A circulating hemocyte was found in the lumen of AMA (arrow head). **h** In the horizontal HPT tissue section different cell types were observed; active precursor- (green arrow head), undergoing mitotic-nuclei- (anaphase) (yellow arrow head), early mature eosinophilic cytoplasm (red arrow head), and condensed euchromatic hemocytes (white arrow head). **i** Below the arterial layer, the longitudinal HS is found to which the mature hemocytes are released into. Large euchromatic nucleate cells are observed in each HPT lobule (arrows). **j** Early mature hemocytes which contain eosinophilic cytoplasm were found in the area immersed with ink (narrow HS). *D, dorsal; V, ventral; R, right; L, left; M, medial; L, lateral; CT, connective tissue; Vs, Vessel; A, artery; M; muscle; Ed, endothelium.*

**Supplementary Fig. S2**

**
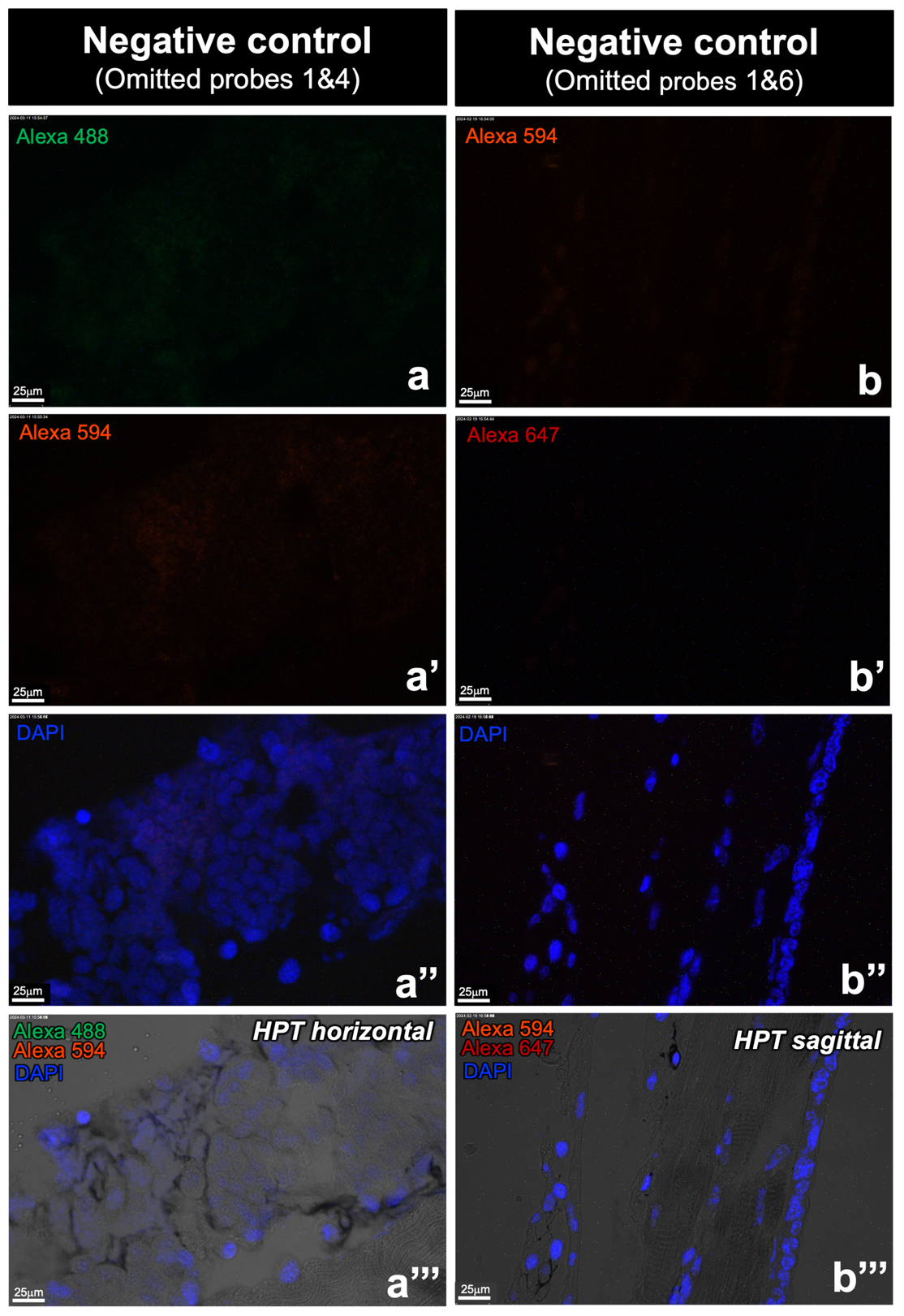
**

**Fig. S2** Fluorescent microscopic photographs of negative controls without probes; probe **a-a”’** for probes type 1 and 4, and (**b-b”’**) for type 1 and 6.

**Supplementary Fig. S3**

**
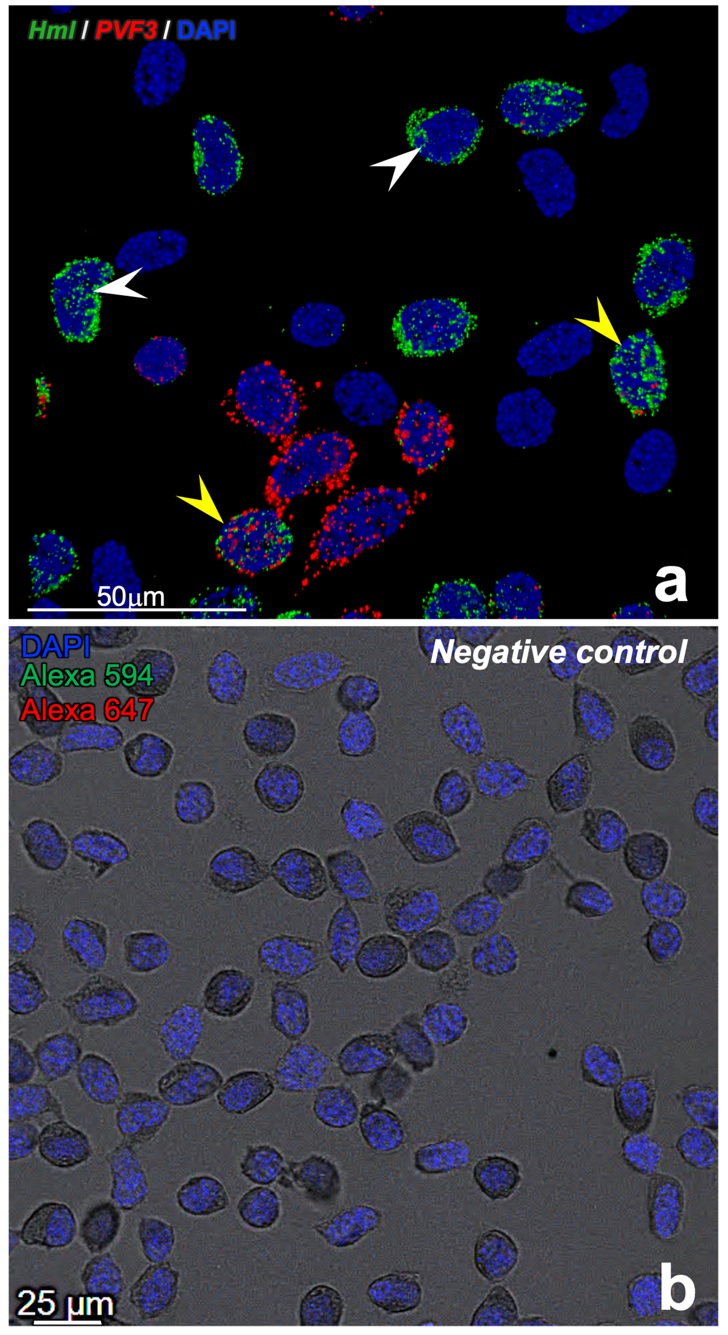
**

**Fig. S3** Higher magnification of figure **6j**. **a** Z-stacking 3D rendered confocal picture showing RNA-FISH localization of *Hml* (green) and *PVF3* (red) in hemocytes. Four cell types were observed; most *PVF3* positive cells did not express *Hml*, a few cells expressed *Hml* and *PVF3* (yellow arrow head), while most *Hml* positive cells (white arrow head) did not express *PVF3*, some cells did not express either *Hml* or *PVF3* (only DAPI stained cell). **b** Negative controls without probes.

**Supplementary Fig. S4**

**
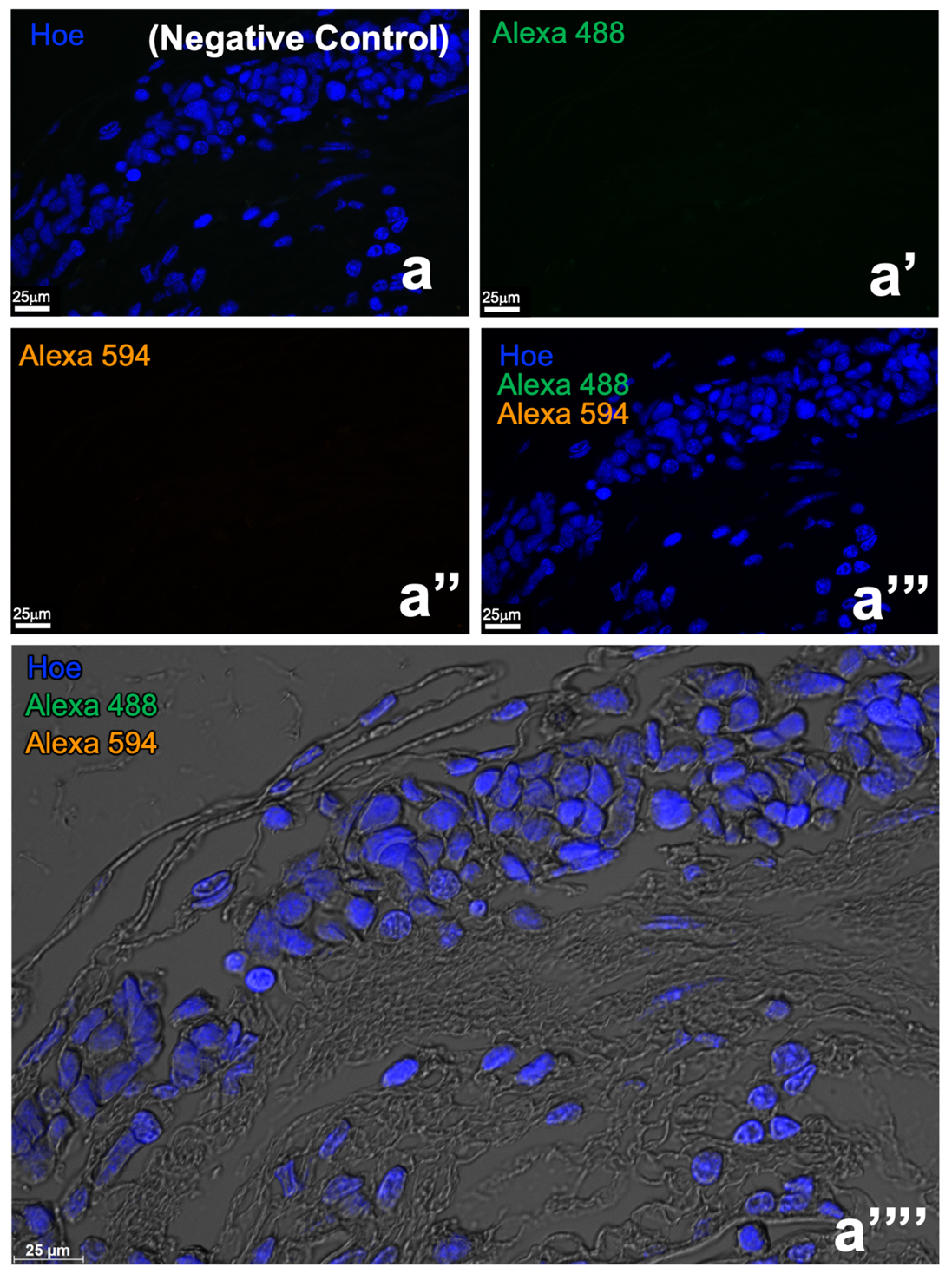
**

**Fig. S4** Fluorescent microscopic photographs of negative control (omitted primary antibodies) of APC tissue without primary antibodies; **a** Hoechst 33258 for nuclear staining, **a’-a’’** no positive signals from Alexa Fluor 488 and 594 channels, **a’’’** merged all channels, and **a””** merged with DIC.

**Supplementary Fig S5**

**
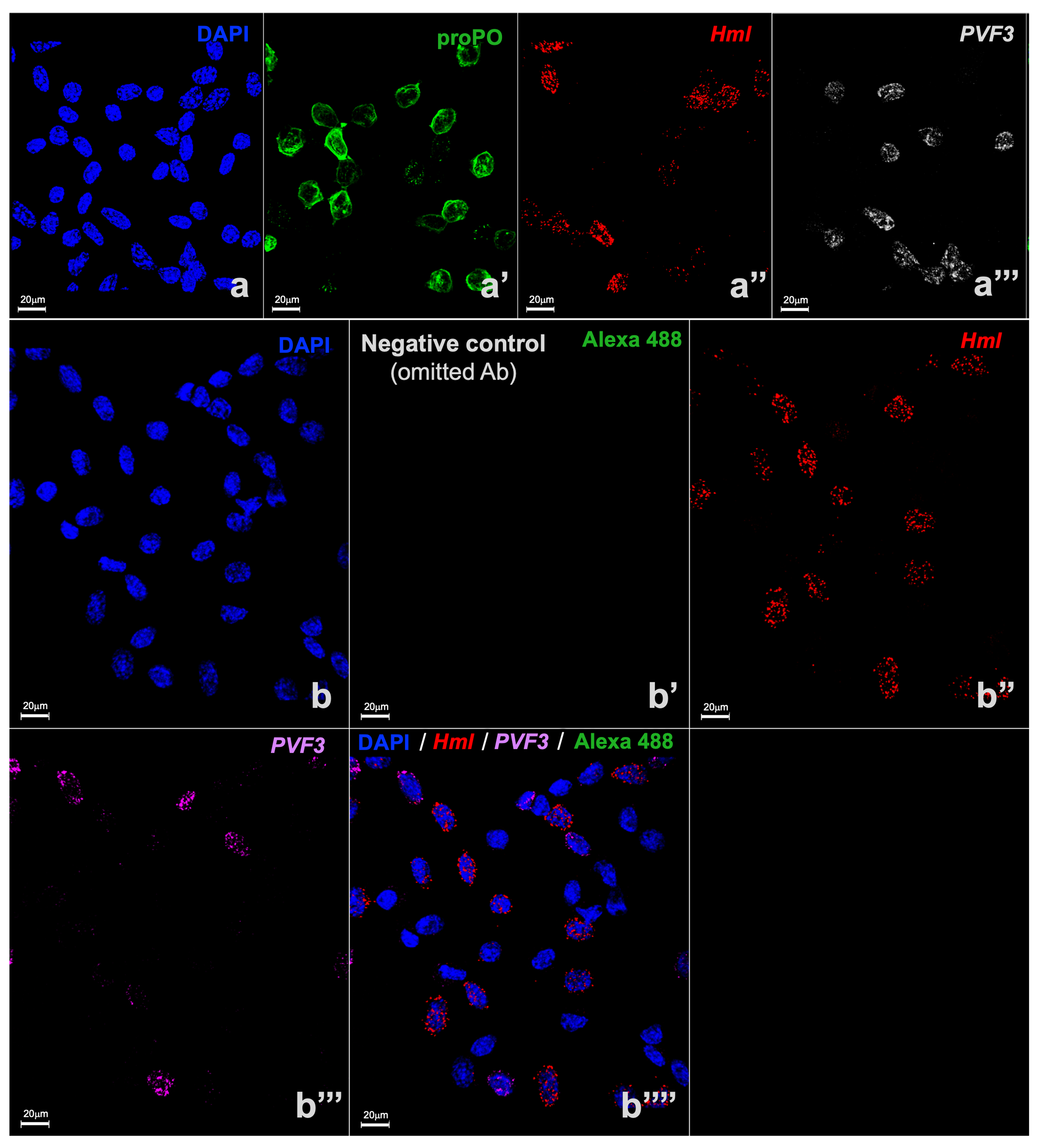
**

**Fig. S5 a**-**a’’’** Co-localization of proPO protein and RNA-FISH specific to *Hml* and *PVF3* transcripts as shown in fig. **8f** and here shown in separate channels. **b**-**b””** Fluorescent signal from separated channels of the negative control group without primary antibody to proPO.
